# Histone H1 Plays a Role in Heterochromatin Formation and VSG Expression Site Silencing in *Trypanosoma brucei*

**DOI:** 10.1371/journal.ppat.1003010

**Published:** 2012-11-01

**Authors:** Megan L. Povelones, Eva Gluenz, Marcin Dembek, Keith Gull, Gloria Rudenko

**Affiliations:** 1 Division of Cell and Molecular Biology, Imperial College London, South Kensington, London, United Kingdom; 2 The Sir William Dunn School of Pathology, University of Oxford, Oxford, United Kingdom; Yale University, United States of America

## Abstract

The African sleeping sickness parasite *Trypanosoma brucei* evades the host immune system through antigenic variation of its variant surface glycoprotein (VSG) coat. Although the *T. brucei* genome contains ∼1500 *VSG*s, only one *VSG* is expressed at a time from one of about 15 subtelomeric *VSG* expression sites (ESs). For antigenic variation to work, not only must the vast *VSG* repertoire be kept silent in a genome that is mainly constitutively transcribed, but the frequency of VSG switching must be strictly controlled. Recently it has become clear that chromatin plays a key role in silencing inactive ESs, thereby ensuring monoallelic expression of *VSG*. We investigated the role of the linker histone H1 in chromatin organization and ES regulation in *T. brucei*. *T. brucei* histone H1 proteins have a different domain structure to H1 proteins in higher eukaryotes. However, we show that they play a key role in the maintenance of higher order chromatin structure in bloodstream form *T. brucei* as visualised by electron microscopy. In addition, depletion of histone H1 results in chromatin becoming generally more accessible to endonucleases in bloodstream but not in insect form *T. brucei*. The effect on chromatin following H1 knock-down in bloodstream form *T. brucei* is particularly evident at transcriptionally silent ES promoters, leading to 6–8 fold derepression of these promoters. *T. brucei* histone H1 therefore appears to be important for the maintenance of repressed chromatin in bloodstream form *T. brucei*. In particular H1 plays a role in downregulating silent ESs, arguing that H1-mediated chromatin functions in antigenic variation in *T. brucei*.

## Author Summary

*Trypanosoma brucei* causes African sleeping sickness, endemic to sub-Saharan Africa. Bloodstream form *T. brucei* is covered with a dense coat of variant surface glycoprotein (VSG). Only one *VSG* is expressed at a time out of a vast repertoire of ∼1500 *VSG*s. The active *VSG* is transcribed in a telomeric *VSG* expression site (ES), and *VSG* switching allows immune evasion. Exactly how monoallelic exclusion of *VSG* ESs operates, and how switching between ESs is mediated remains mysterious, although epigenetics and chromatin structure clearly play a major role. The linker histone H1 is thought to orchestrate higher order chromatin structure in eukaryotes, but its exact function is unclear. We investigated the role of histone H1 in the regulation of antigenic variation in *T. brucei*. We show that histone H1 is associated with chromatin and is required for higher order chromatin structure. Depletion of histone H1 results in derepression of silent *VSG* ES promoters, indicating that H1-mediated chromatin functions in antigenic variation in *T. brucei*.

## Introduction

The African trypanosome *Trypanosoma brucei* is a unicellular parasite causing African sleeping sickness, which is transmitted by tsetse flies in sub-Saharan Africa. As an extracellular parasite of the mammalian bloodstream, *T. brucei* has evolved a sophisticated strategy to antigenically vary its major surface coat protein, variant surface glycoprotein (VSG) [1], [2]. The *T. brucei* genome contains a vast repertoire of silent *VSG* genes and pseudogenes, most of which are located in tandem arrays at subtelomeric locations [3], [4]. The *VSG* repertoire varies in both size and composition between different *T. brucei* strains, with the exact sizes still unclear due to the technical complications of cloning, sequencing and assembling these subtelomeric sequences [5]. However, a conservative estimate proposes that the *T. brucei* 927 strain contains more than 1500 *VSG*s, of which only one *VSG* is expressed at a time [6], [7].

The active *VSG* is located in one of about 15 telomeric *VSG* expression sites (ES). ESs are transcribed by RNA polymerase I (Pol I) [8], [9], which normally exclusively transcribes ribosomal DNA (rDNA) [10]. For antigenic variation to work, it is key that only one *VSG* is expressed at a time, and the extensive repertoire of *VSG*s is kept transcriptionally silent. These restrictions need to operate within the context of a *T. brucei* genome which is primarily organised as very extensive polycistronic transcription units constitutively expressed by Pol II [6], [11]. Although it is unclear how ESs are controlled, it has recently been shown that chromatin remodeling must play a key role in their regulation [12]–[14].

In eukaryotes DNA is packaged into nucleosomes, whereby ∼146 bp of DNA is wrapped around a histone octamer consisting of two histone H2A/H2B dimers and two histone H3/H4 dimers. A linker histone H1 (H1) typically interacts with both the nucleosome and the linker DNA to stabilize higher order chromatin structure [15]. H1 has been shown to be dispensable in several unicellular eukaryotes including yeast and Tetrahymena [16]–[18]. The exact role of H1 has been surprisingly hard to discern despite its association with heterochromatin and proposed function as a general transcriptional repressor [19]–[23]. Knock-out of H1 in *S. cerevisiae*, Tetrahymena or mammalian cells affects transcription of a relatively small subset of genes in these different organisms, and does not have a major effect on global transcription [24]–[26]. In addition, yeast cells lacking histone H1 demonstrate genomic instability, most likely due to increased homologous recombination (HR) in its absence [27].

The chromatin of *T. brucei* has several unusual properties. The core histones of *T. brucei* are divergent compared with those of higher eukaryotes, particularly at the N-termini which can be post-translationally modified [28]–[30]. In addition, *T. brucei* chromatin has a more open conformation, does not form 30-nm fibres *in vitro*, and chromosomes fail to condense prior to nuclear mitosis [31]. These characteristic features of *T. brucei* chromatin are typically influenced by the linker histone H1 in other eukaryotes arguing that *T. brucei* H1 could play a different role [15].

Histone H1 proteins in *T. brucei* are distinct from those in other eukaryotes, in that they lack the central globular domain thought to be responsible for interaction with the nucleosome [32]. Instead, they consist of a single domain corresponding to the C-terminal domain of H1 proteins in higher eukaryotes [33]. This C-terminal domain has been shown to be essential for both the DNA binding and chromatin compaction functions of H1 [33]–[36]. Single-domain linker histones are also found in other kinetoplastid species, as well as in Tetrahymena and in eubacteria [21]. Importantly, this type of truncated H1 protein has been shown to affect chromatin structure through a mechanism of DNA compaction which may be mechanistically distinct from higher eukaryotes [16], [37]–[39].

We have investigated the role of histone H1 in the regulation of antigenic variation in *T. brucei*, as well as in the maintenance of higher order chromatin states. Depletion of histone H1, while having a minimal effect on cell growth, causes significant changes in chromatin structure. H1 knock-down resulted in increased sensitivity to endonucleases in bloodstream *T. brucei*, but not in the procyclic form of the parasite which replicates in the midgut of the insect vector and does not express VSG. In particular, reduced levels of H1 result in the formation of a more open chromatin structure in the vicinity of silent ES promoters in bloodstream *T. brucei*, which is correlated with an increase in transcription at silent ESs. Knockdown of a number of chromatin proteins in *T. brucei* results in disruption of *VSG* ES silencing or VSG switching [40]–[43]. We now show that histone H1 plays a role in regulating ES repression, thereby providing a link between this linker histone and the process of antigenic variation in bloodstream form *T. brucei*.

## Results

### Histone H1 proteins are associated with chromatin in *T. brucei*

**Figure 1:**
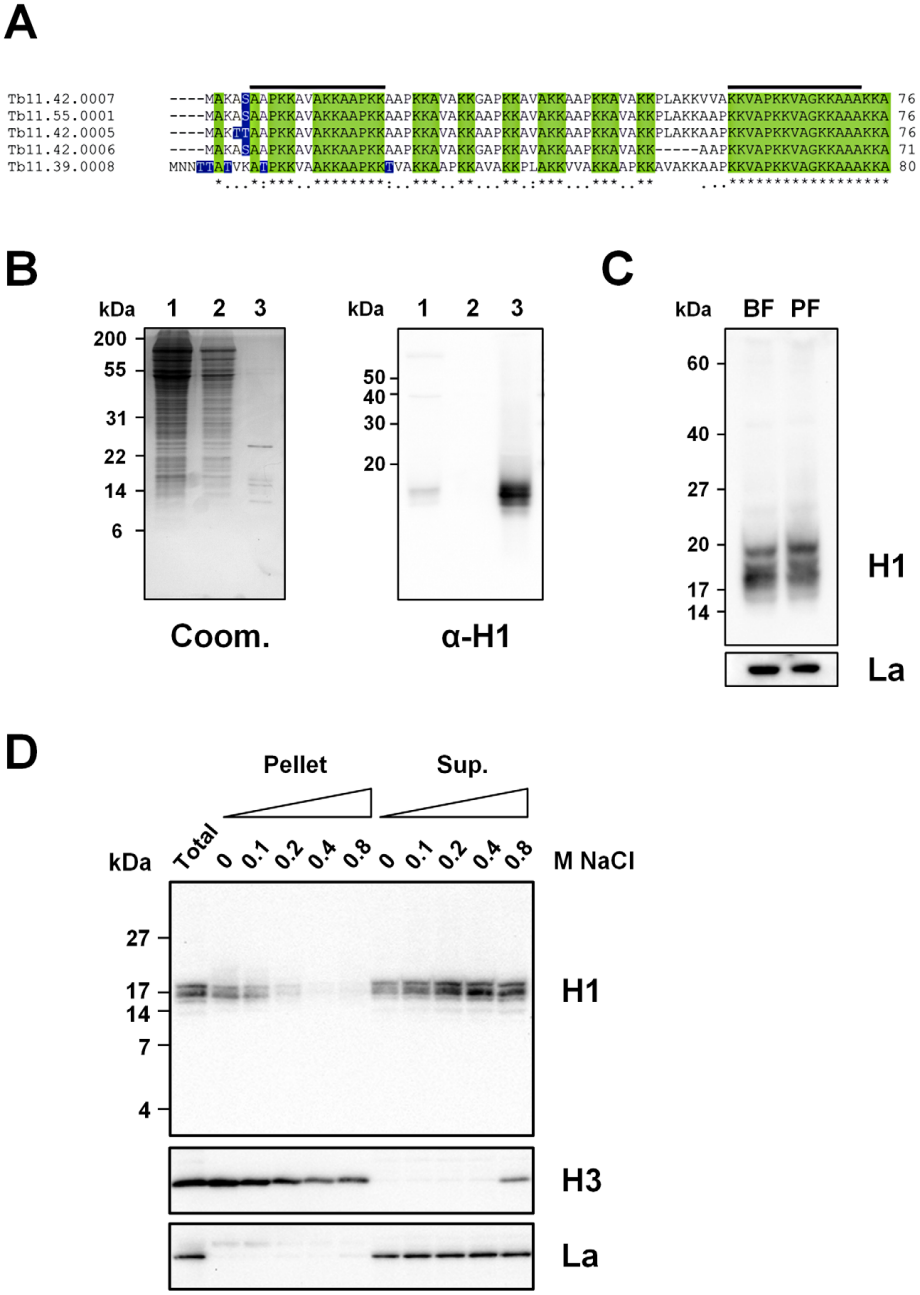
Histone H1 proteins in *T. brucei*. **A.** Alignment of predicted histone H1 proteins from the genome sequence of *T. brucei* 927 [3]. Green boxes indicate identical residues. Blue boxes indicate N-terminal serine/threonine residues, which may be targets for phosphorylation. Black bars indicate the peptides used for *T. brucei* histone H1 antibody production. **B.** Perchloric acid extraction of histone H1 proteins in *T. brucei*. Lanes show total *T. brucei* cell lysate (lane 1), supernatant after lysis by douncing (lane 2), and the fraction that has been extracted by perchloric acid treatment (lane 3). The left panel shows the Coomassie stained SDS-PAGE gel, and the right panel shows the Western blot of a Laemmli (glycine-based) SDS-PAGE gel reacted with an anti-histone H1 antibody. The sizes of marker proteins are indicated in kiloDaltons (kDa). **C.** Expression of histone H1 proteins in bloodstream form (BF) *T. brucei* HNI 221+ and procyclic form (PF) *T. brucei* 221BsrDsRed. Cells were lysed in SDS-PAGE sample buffer and proteins were resolved on Tris-tricine SDS-PAGE gels which were run for ∼5 hours for optimal resolution. The Western blot was probed with the anti-histone H1 antibody. An antibody against the La RNA binding protein is used as a loading control. Note that different protein standards were used for Tris-glycine and Tris-tricine SDS-PAGE gels, and that proteins migrate slightly differently using these different methods of electrophoresis. **D.** The *T. brucei* linker histone H1 has less affinity for chromatin than the core histone H3. BF *T. brucei* cells were lysed in 1% Triton X-100 in the presence of increasing amounts of NaCl. Pellet and supernatant (Sup.) fractions were analysed by Tris-tricine SDS-PAGE followed by Western blot analysis monitoring for histone H1, the core histone H3 and the La RNA binding protein. Total indicates total lysate. The concentration of NaCl (M) added during cell lysis is indicated above the relevant lane. The size of marker proteins in kiloDaltons is indicated.

The *T. brucei* 927 genome sequence contains five predicted histone H1-like genes arranged in two clusters on chromosome 11 (Fig. 1A) [3], [44]. The predicted *T. brucei* H1 proteins are small (7–8 kDa), very basic proteins (pI of ∼12) with at least one serine or threonine residue at the N-terminus. We raised antibodies using peptides which theoretically should allow recognition of all five *T. brucei* histone H1 isoforms. Histone H1 proteins have characteristic biochemical properties including the ability to be extracted with perchloric acid [31], [37], [45]. We therefore isolated H1 proteins from procyclic form *T. brucei* using perchloric acid extraction. Several proteins were enriched, and Western blot analysis confirmed that a subset reacts with our H1 antibody (Fig. 1B). We compared the H1 species present in either bloodstream or procyclic form *T. brucei* using Tricine-SDS-PAGE gels (Fig. 1C). We observed a slightly different histone H1 banding pattern in the two *T. brucei* life-cycle stages [45], possibly due to post-translational modifications of different H1 isoforms, as has been found in other organisms [46]. Determining the significance of these different banding patterns is complicated by the fact that our histone H1 antibody may have different affinities for different isoforms and post-translationally modified versions of H1. However, it is interesting to note that other laboratories have observed similar H1 banding patterns using non-antibody-dependent methods [45].

We next determined the association of *T. brucei* histone H1 with chromatin in bloodstream form *T. brucei*, by isolating chromatin containing fractions in increasing concentrations of NaCl (Fig. 1D) [42]. As expected, the core histone H3 remained associated with the chromatin fraction in all but the highest salt concentration (0.8 M NaCl), while the RNA binding protein La did not associate with the chromatin fraction [47]. Histone H1 proteins were detected in the chromatin fractions at salt concentrations of up to 0.2 M NaCl, indicating association with DNA. However, some proportion of H1 is soluble in the absence of NaCl. This suggests that as expected, *T. brucei* linker histone H1 has a weaker affinity for chromatin than the core histones [37], [45]. Similar results were obtained using procyclic form lysates (Sup. Fig. S1).

**Figure 2:**
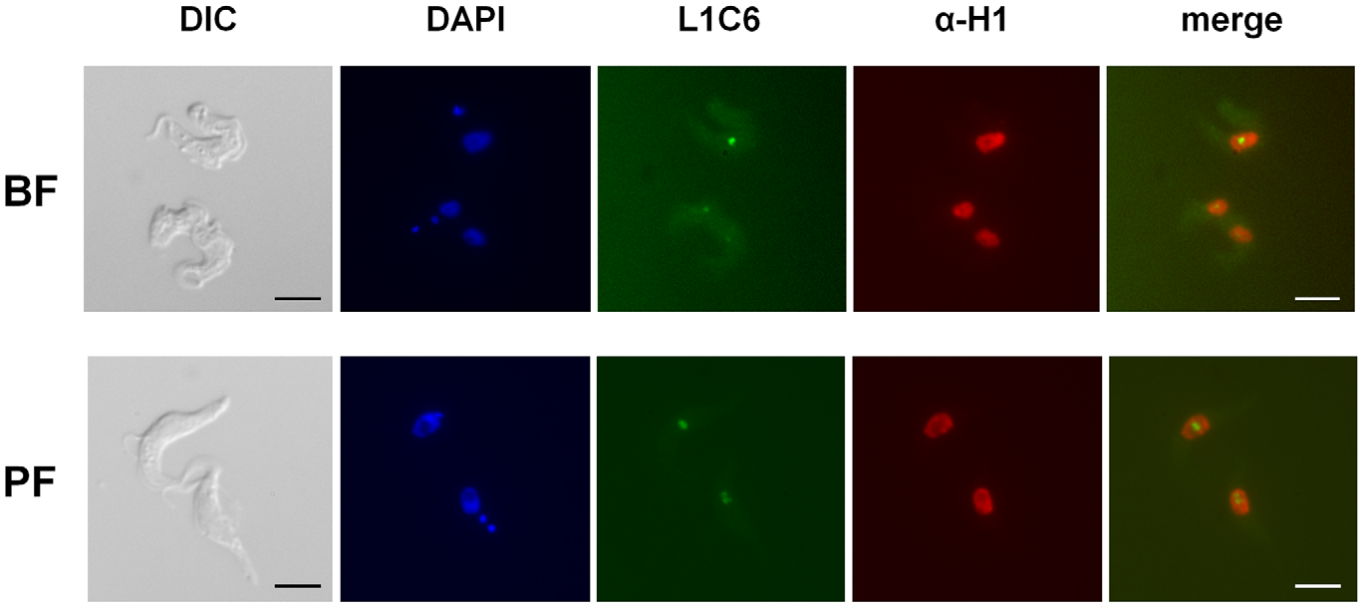
Nuclear localization of histone H1 in *T. brucei*. BF *T. brucei* HNI 221+ and PF *T. brucei* 221BsrDsRed cells were fixed and analysed by immunofluorescence microscopy using anti-histone H1 antibodies (αH1). The monoclonal L1C6 antibody was used to visualise the nucleolus, and a differential interference contrast (DIC) image is shown. Scale bar is 5 µm.

We determined the subcellular localisation of histone H1 using immunofluorescence microscopy, and identified a strong nuclear signal in both bloodstream and procyclic form *T. brucei* (Fig. 2). Notably, H1 appeared to be depleted from the nucleolus. We confirmed that the nucleolus was indeed accessible to antibodies by co-staining with the monoclonal L1C6 antibody which specifically identifies the nucleolus [48]. This observed relative depletion of H1 from the nucleolus is presumably a consequence of the extremely high rates of transcription of the ribosomal DNA (rDNA) in this location.

### Histone H1 is depleted from highly transcribed regions of the *T. brucei* genome

**Figure 3:**
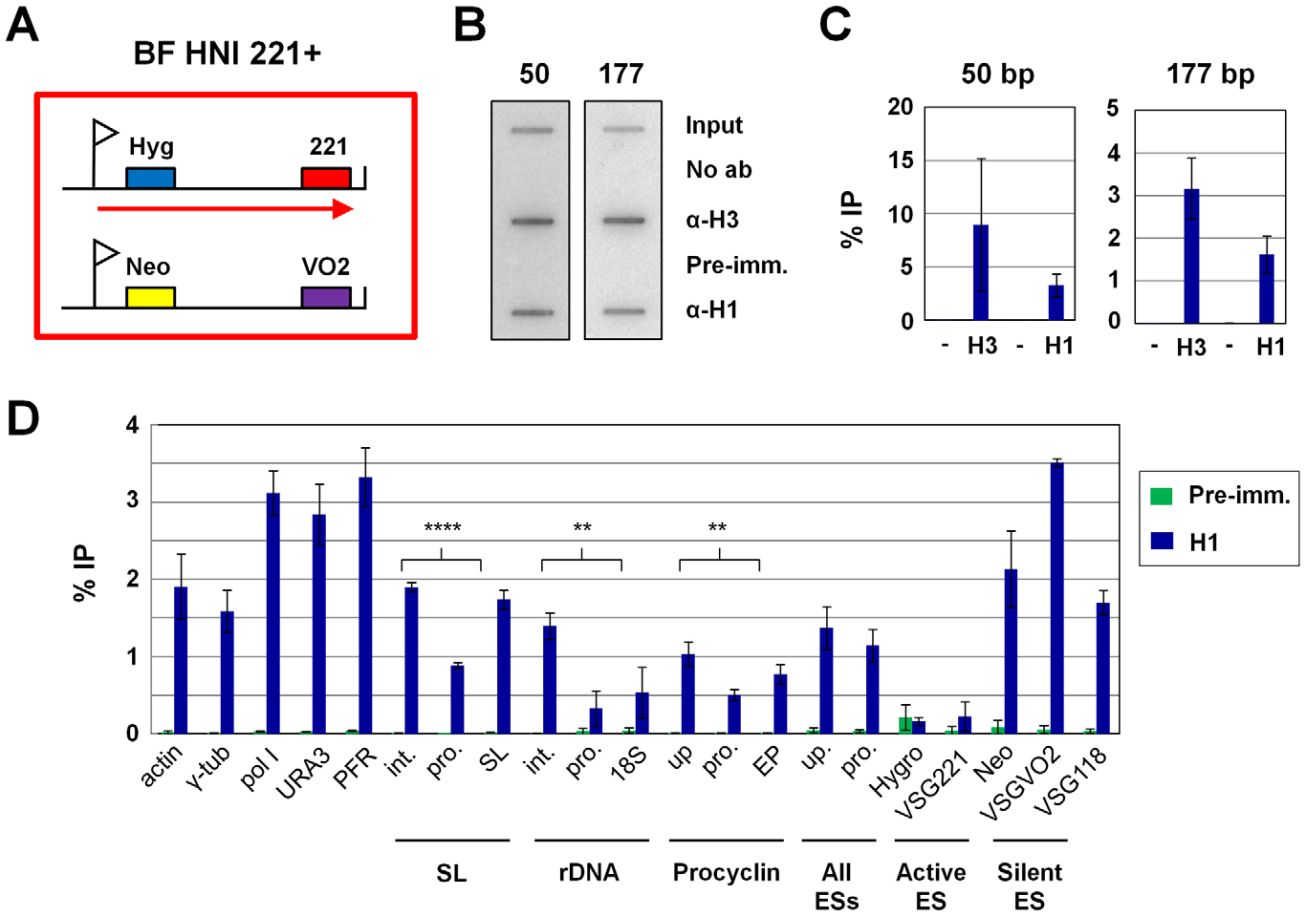
Distribution of histone H1 in the *T. brucei* genome. **A.** Schematic of the BF *T. brucei* HNI 221+ cell line used for ChIP experiments indicated with a large red box. *VSG* expression sites (ESs) containing the hygromycin (Hyg) or neomycin (Neo) resistance genes, as well as the telomeric *VSG221* (221) and *VSGVO2* (VO2) genes are indicated. The ES promoters are indicated with flags, and transcription of the active *VSG221* ES with an arrow. **B.** Representative slot blots of chromatin immunoprecipitation (ChIP) samples showing the presence of histone H3 or histone H1 on characteristic 50 bp repeat sequences found flanking ESs, or 177 bp repeats comprising the bulk of the *T. brucei* minichromosomes. Experiments were performed with no antibody (No ab) or pre-immune serum (Pre-imm.) from the rabbit used to produce the histone H1 antibody as negative controls (−). For each immunoprecipitated sample, 10% of the ChIP material was loaded on a slot blot and compared to 0.1% of the total input. **C.** Quantitation of material immunoprecipitated (% IP) using anti-histone H3 (H3) or anti-histone H1 (H1) in the slot blots shown in panel B. Bars show the mean of three experiments with standard deviation indicated with error bars. Two negative controls (−) were used: no antibody, or the pre-immune serum. **D.** Distribution of histone H1 within the genome of BF *T. brucei* as determined using qPCR analysis of ChIP material. The bars indicate the amount immunoprecipitated (% IP) using the anti-histone H1 antibody (H1) or pre-immune serum (Pre-imm.) as a control, with the standard deviation in three experiments indicated with error bars. Statistically significant (P<0.01) amounts of histone H1 were immunoprecipitated in all regions with the exception of the rDNA promoter, 18S rRNA, hygro and *VSG221* genes (Supplemental Fig. S3). The RNA polymerase II (Pol II) transcribed regions analysed are the actin, γ-tubulin (γ-tub), RNA polymerase I large subunit (pol I), *URA3*, paraflagellar rod protein B (PFR) and spliced leader (SL) gene loci. The SL intergenic region (int.), promoter region (pro.), or the SL gene itself (SL) are indicated. The ribosomal DNA (rDNA) regions analysed include the rDNA intergenic region (int.), promoter (pro.) or the 18S rDNA gene (18S). The EP procyclin locus analysed includes the region upstream of the EP promoter (up.), the promoter (pro.), or the EP procyclin gene (EP). Higher levels of histone H1 were immunoprecipitated upstream of the promoters compared with at the promoter regions themselves, with the statistical significance indicated with asterisks (** indicates P<0.01, and **** indicates P<0.0001). ES sequences analysed include a region immediately upstream of the ES promoter (up.) as well as at the promoter itself (pro.). These primer pairs can be expected to recognise most if not all ESs. Sequences specific for the active *VSG221* ES include the hygromycin resistance gene (Hygro) as well as *VSG221.* Sequences present in the silent *VSGVO2* ES include the neomycin resistance gene (Neo) and *VSGVO2*. Note that the *VSGVO2* primers detect both the telomeric ES located *VSGVO2* gene, as well as the chromosome-internal copy of *VSGVO2*. *VSG118* is found in the silent *VSG* basic copy arrays.

We subsequently used chromatin immunoprecipitation (ChIP) to investigate the genomic distribution of H1 in *T. brucei* using either our affinity-purified H1 antibody, or a histone H3 antibody as a positive control (Fig. 3). We first determined the distribution of H1 on two extensive nontranscribed regions of the *T. brucei* genome. The 50 bp simple sequence repeats form large nontranscribed arrays flanking all known *VSG* ESs (Fig. 3B, 3C) [49]. H1 is significantly enriched here, as well as on the 177 bp repeats which comprise the bulk of the transcriptionally inactive *T. brucei* minichromosomes [50].

We subsequently used quantitative PCR (qPCR) to determine H1 distribution on different transcription units in bloodstream form *T. brucei* (Fig. 3D). Statistically significant levels of H1 were present in RNA polymerase II (Pol II) transcribed regions, including the actin, γ-tubulin, RNA polymerase I large subunit (Pol I), *URA3*, paraflagellar rod protein B (PFR) and spliced leader (SL) gene loci (Fig. 3D). Significantly less H1 was found at the SL promoter, compared with at the region upstream of the SL gene (P<0.0001). Similarly, H1 was enriched on the non-transcribed rDNA spacer compared with the rDNA promoter or the 18S rRNA gene (P = 0.003, or P = 0.0159 respectively). A similar pattern was observed at the procyclin locus, where more H1 was bound upstream of the EP procyclin promoter compared with at the EP procyclin promoter itself (P = 0.006). Similar results were found in procyclic form *T. brucei* (Sup. Fig. S2).

H1 was found associated with ESs using qPCR primers that detect all ESs. However using primers specific for either the *VSG221* or *VSGVO2* ES, we showed that H1 is depleted from the active *VSG221* ES (hygromycin and *VSG221* genes) compared with the silent *VSGVO2* ES (neomycin and *VSGVO2*). Note that the primer pair for *VSGVO2* will also detect an additional non-ES located copy of *VSGVO2*. This H1 distribution is similar to that of the core histones H2A, H3 and H4 [13], [14]. H1 is also enriched on the nontranscribed *VSG118* gene located at a chromosome internal *VSG* basic copy array. The distribution of H1 in procyclic form *T. brucei* is similar, with the exception that H1 does not appear to be depleted from the SL promoter (Sup. Fig. S2). It is interesting to note that the Pol II transcribed polycistronic arrays are not as depleted of H1 as the active ES, implying that a completely open chromatin structure may not be required for efficient transcription of these regions.

### Depletion of histone H1 leads to a growth defect and changes in chromatin structure

**Figure 4:**
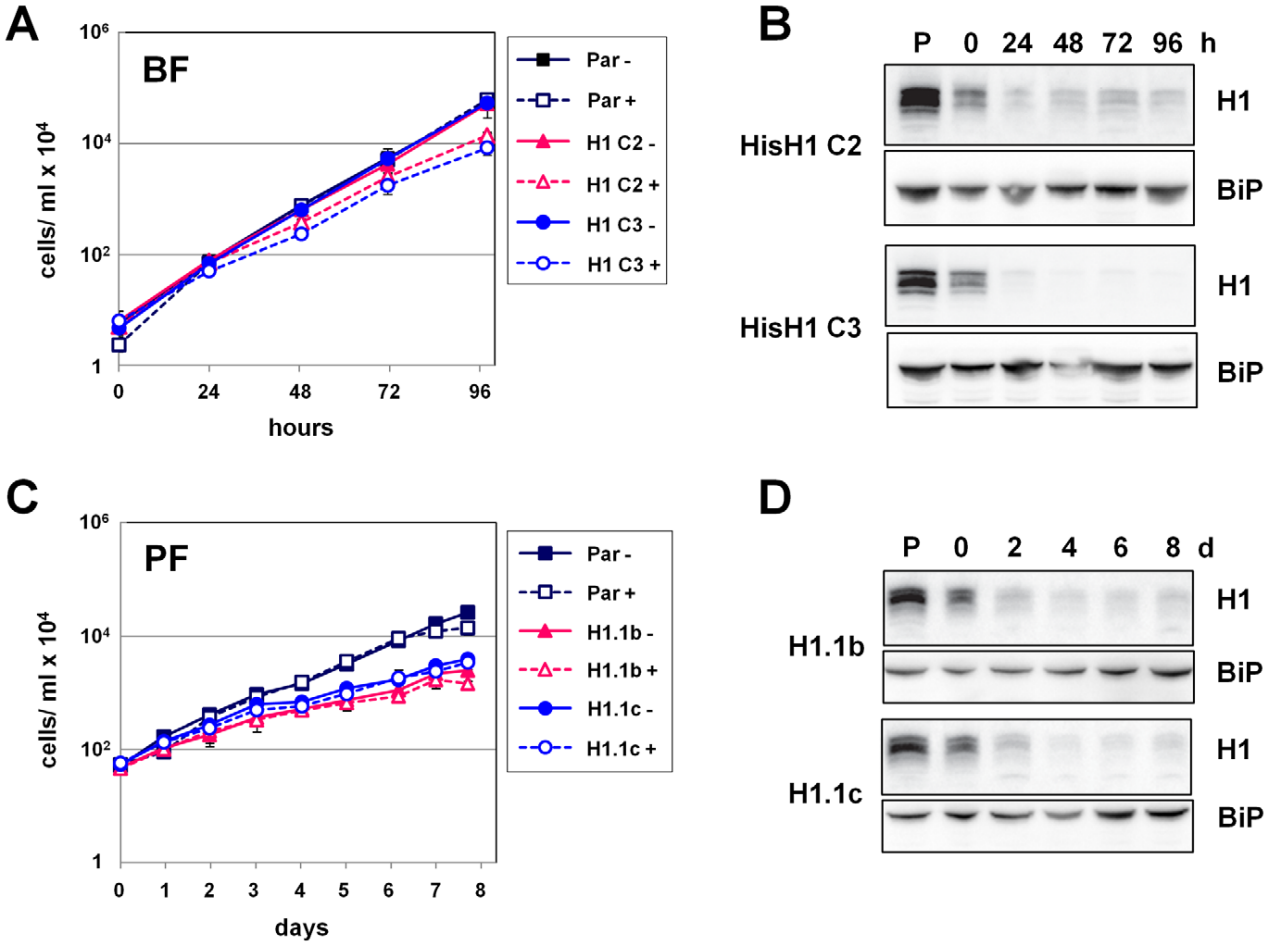
RNAi-mediated depletion of histone H1 proteins results in a moderate reduction in growth rates. **A.** Growth curves of parental BF *T. brucei* or two independent BF *T. brucei* RYT3-H1 clones in the presence (+) or absence (−) of tetracycline to induce histone H1 RNAi. The mean of three experiments is shown with the standard deviation indicated with error bars. In each case, the cell number was multiplied by the dilution factor to obtain a value for cumulative cell growth. **B.** Western blot analysis of Tris-tricine gels showing knockdown of histone H1 in two independent BF *T. brucei* RYT3-H1 histone H1 RNAi clones compared with the parental (P) cell line. Histone H1 RNAi was induced with tetracycline for the time indicated in hours (h). BiP is shown as a loading control. **C.** Experiment similar to as shown in panel A. performed in PF *T. brucei* cells. **D.** A similar experiment as shown in panel B. performed using PF *T. brucei* cells. Histone H1 RNAi was induced with tetracycline for the time indicated in days (d).

To study the function of histone H1 proteins in *T. brucei*, we performed inducible H1 RNAi using a construct that would be expected to target all five H1 genes. We cloned two fragments corresponding to different regions of the polymorphic H1 gene arrays in tandem into the p2T7-177 vector allowing tetracycline inducible H1 RNAi [51]. In bloodstream form *T. brucei*, a small but reproducible reduction in growth was observed after the induction of H1 RNAi (Fig. 4A). Western blot analysis revealed that the different H1 proteins were maximally depleted by 24 hours (Fig. 4B). However H1 levels were already reduced in the uninduced (0 h) samples compared with in the parental *T. brucei* line, presumably as a consequence of leaky transcription of the RNAi construct [52]. In procyclic form *T. brucei*, cells containing the H1 RNAi construct grew slower than the parental line even in the absence of tetracycline, again presumably due to leaky transcription (Fig. 4C). Western blot analysis of procyclic form *T. brucei* after the induction of H1 RNAi showed that H1 depletion was maximal at 96 hours (Fig. 4D). However, the additional H1 knockdown observed after induction of H1 RNAi in procyclic form cells had no further effect on growth. These RNAi experiments provide evidence for specificity of our anti-histone H1 antibody, as the putative histone H1 proteins detectable by Western blot indeed decreased after the induction of H1 RNAi (Fig. 4B and 4D). In addition, RNAi-mediated depletion of histone H1 leads to a loss of H1 nuclear staining as observed using immunofluorescence microscopy (data not shown).

**Figure 5:**
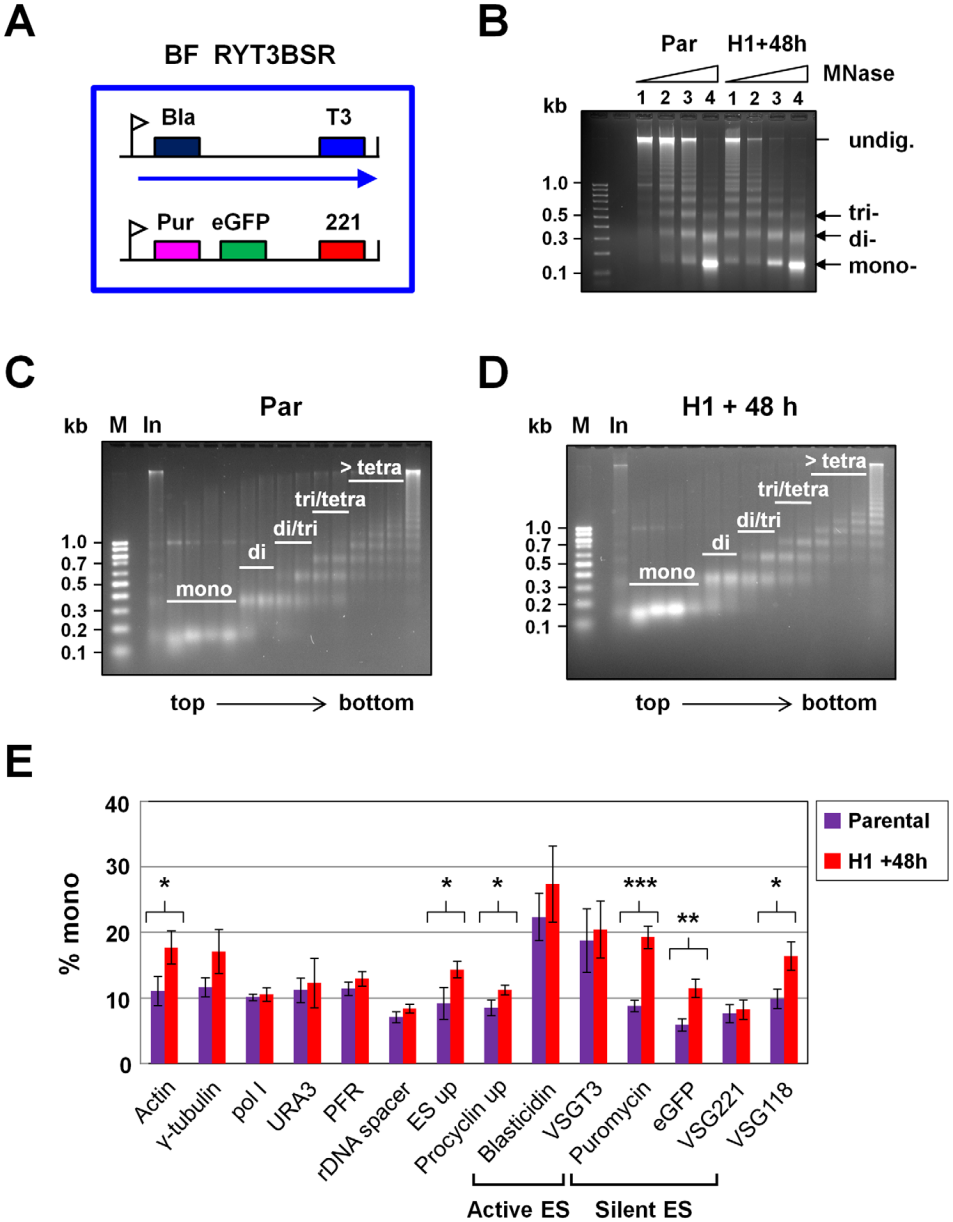
Chromatin structure in the presence of reduced histone H1. **A.** Schematic of the *T. brucei* BF RYT3-H1 cell line in which micrococcal nuclease (MNase) sensitivity experiments were performed after the induction of histone H1 RNAi. The large blue box indicates the BF *T. brucei* cell, with a blasticidin (Bla) gene integrated in the active *VSGT3* ES (T3), and the puromycin (Pur) resistance gene and *eGFP* integrated immediately behind the promoter of the silent *VSG221* ES. The expression site (ES) promoters are indicated with white flags, and ES transcription with an arrow. A construct allowing tetracycline inducible transcription of histone H1 RNAi (H1) from opposing T7 promoters (facing arrows) has also been introduced into these cells using a hygromycin resistance gene (Hyg) transcribed from an rDNA promoter (black flag). **B.** Parental (Par) BF *T. brucei* RYT3BSR cells and RYT3-H1 cells in which histone H1 had been knocked down by the induction of RNAi for 48 h (H1+48 h) were permeabilized and incubated with increasing concentrations of MNase (Lane 1 = 0.0625 units MNase, lane 2 = 0.125 units, lane 3 = 0.25 units and lane 4 = 0.5 units. Isolated DNA was visualized on ethidium bromide-stained agarose gels, and a characteristic ladder pattern was observed. Products corresponding to DNA that had been bound to mono-, di, or trinucleosomes, as well as undigested DNA (undig.) are indicated. DNA sizes are indicated in kilobases (kb) on the left. **C.** Sucrose gradient fractionation of MNase-digested chromatin from BF *T. brucei* RYT3BSR cells (Par). Chromatin in permeabilized cells was subjected to MNase treatment and then loaded onto a 5–30% sucrose step gradient (input). After centrifugation, fractions were removed, and DNA isolated. Fractions 11–24 are shown with top to bottom of the gradient indicated with an arrow. Fractions 1–10 contained very little DNA and are not shown. Fractions containing mono-, di-, di-/tri-, tri-/tetra-, and >tetranucleosomes are indicated with white bars. These fractions were used to create five pools of DNA which were used as templates for qPCR. **D.** BF *T. brucei* cells in which histone H1 RNAi had been induced for 48 hours. Chromatin in permeabilized cells was subsequently subjected to MNase treatment and was further analysed as described in panel C. **E.** Induction of histone H1 RNAi for 48 hours affects the distribution of various *T. brucei* genomic regions in the mononucleosomal fractions containing open chromatin. Results show qPCR analysis of fractionated, MNase-treated DNA. The amount of each target detected in the mononucleosome pool is plotted as a percentage of the total amount of target detected in all pools. Genomic regions analysed are as indicated in Fig. 3 panel D. The mean of three independent experiments is shown with error bars indicating standard deviation. Histone H1 knock-down resulted in a statistically significant increased distribution of various regions in the mononucleosomal fraction, with asterisks indicating statistical significance (*, P<0.05; **, P<0.01; ***, P<0.001).

We next investigated the role of H1 in the maintenance of *T. brucei* chromatin structure using micrococcal nuclease (MNase). MNase preferentially digests DNA in between nucleosomes, such that an open chromatin structure is more readily cleaved than closed chromatin. Bloodstream form parental *T. brucei* and cells in which H1 RNAi had been induced were treated with increasing concentrations of MNase (Fig. 5A). DNA was analysed, and characteristic ladders corresponding to mono-, di-, and tri-nucleosomal species were observed (Fig. 5B) [14]. Chromatin from cells in which H1 RNAi had been induced for 48 hours was reproducibly more sensitive to MNase digestion compared with that from the parental line (compare lanes 1 in Fig. 5B). This indicates that knockdown of H1 results in DNA becoming more accessible to digestion by MNase, indicating that H1 helps maintain chromatin in a closed state. Interestingly, this increase in the accessibility of chromatin to MNase digestion after H1 knockdown was not observed in procyclic form *T. brucei* (Sup. Fig. S4). Possibly, the chromatin structure in procyclic form *T. brucei* is already more open than that in bloodstream form *T. brucei* [45], thereby minimising the impact of H1 depletion.

We next investigated the effect of H1 knockdown on chromatin structure at different genomic regions in bloodstream form *T. brucei* in more detail. We treated permeabilized cells with MNase and fractionated the different nucleosomal species on sucrose gradients (Fig. 5C, D). Again, we observed a dramatic increase in DNA in the mononucleosomal fraction after knockdown of H1 (Fig. 5D). We next pooled DNA fractions according to whether they primarily contained mono-, di-, di-/tri-, tri-/tetra-, or >tetranucleosomes. We determined the presence of different *T. brucei* genomic regions in the various fractions using qPCR, and expressed the amount of each qPCR target detected in each pool as a percentage of the total amount amplified in all pooled fractions (Fig. 5E). This allowed us to determine changes in the distribution of different genomic regions across the different chromatin types, while correcting for any differences in the amount of material loaded.

As expected, sequences in the actively transcribed *VSGT3* ES (blasticidin resistance and *VSGT3* genes) were enriched in the mononucleosomal fraction compared with sequences in the transcriptionally silent *VSG221* ES (puromycin resistance, *eGFP* or *VSG221* genes) (18–22% of total compared with 6–8%, respectively) (Fig. 5E). This indicates that as expected, active ESs have a relatively open chromatin structure, and are preferentially digested down to mononucleosomes [14]. Interestingly, the Pol II-transcribed actin, γ-tubulin, Pol I large subunit, PFR and *URA3* regions, while transcriptionally active, appeared to have a less open chromatin state (i.e not enriched in the mononucleosomal fraction) than Pol I transcribed regions. This is similar to as observed for transcriptionally inactive sequences such as *VSG118* (Fig. 5E), and is consistent with the distribution of the core histones across these regions as was previously determined [14].

After blocking H1 synthesis for 48 hours, several regions of the *T. brucei* genome became more accessible to MNase. This effect was most pronounced at the promoter of the silent *VSG221* ES (puromycin resistance and *eGFP* genes) (P = 0.0007 and P = 0.0043 respectively) (Fig. 5E). H1 knockdown also resulted in a significant increase in MNase accessibility at the Pol II transcribed actin genes, as well as at the nontranscribed *VSG118* in the *VSG* basic copy array and the regions immediately upstream of the ES and procyclin promoters (P values<0.05). These data indicate that depletion of H1 results in a general opening of chromatin structure, however this effect is particularly clear at the silent ES promoters. Although depletion of H1 caused changes in chromatin structure upstream of the procyclin promoter, we did not detect increases in the levels of procyclin transcript following H1 knockdown (Sup Fig. S5).

**Figure 6:**
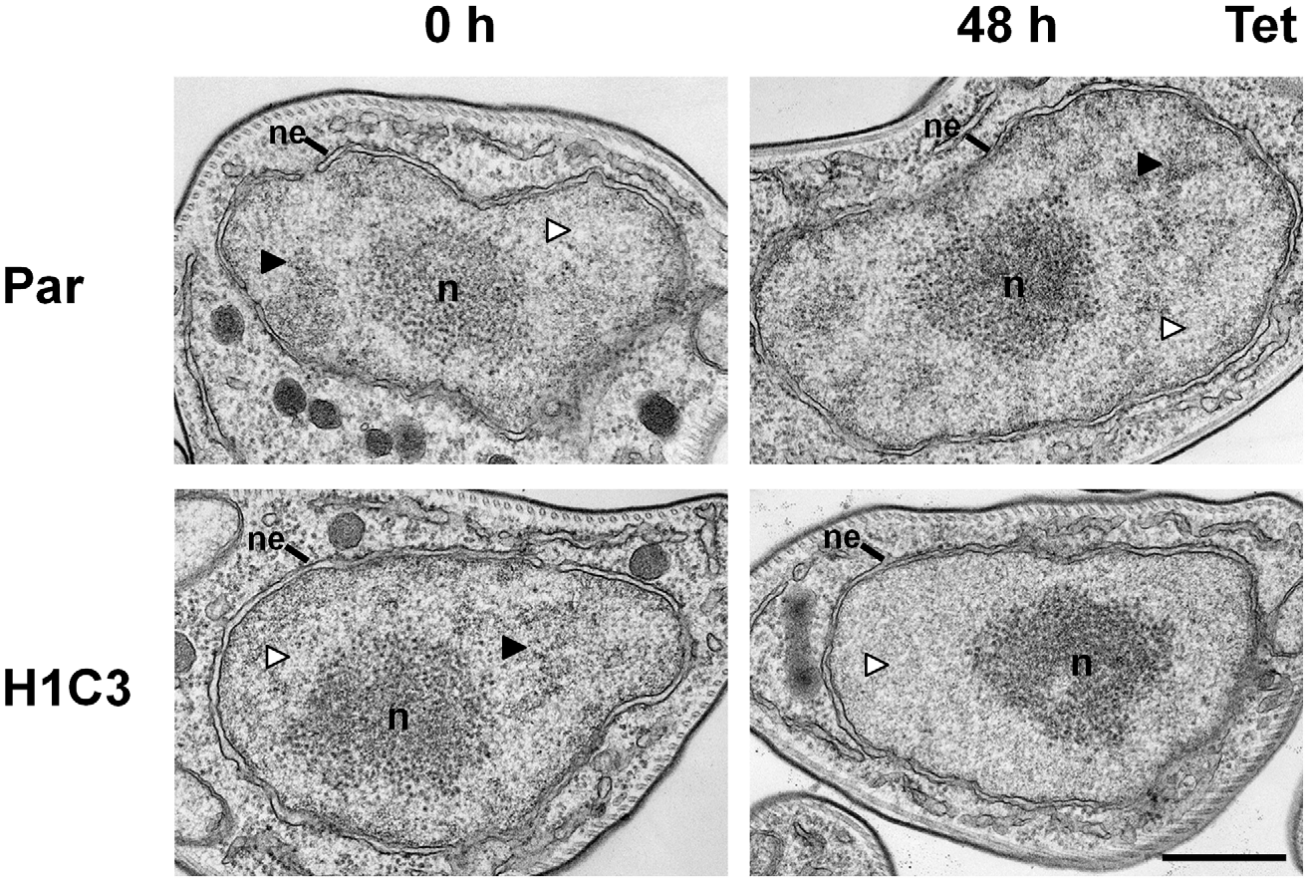
Alteration of nuclear ultrastructure in bloodstream form *T. brucei* after depletion of histone H1. The bloodstream form *T. brucei* RYT3BSR parental (Par) and the RYT3-H1C3 histone H1 RNAi cell line (H1C3) were cultured in the presence (48 h) or absence (0 h) of tetracycline (Tet) and analysed using transmission electron microscopy (TEM). Images show representative sections of nuclei that include the nucleolus (n) and the nuclear envelope (ne). The nuclei of parental *T. brucei* or RYT3-H1C3 lines before the induction of histone H1 RNAi have normal nuclear staining, where the nucleoplasm is divided into domains of greater electron density (black arrowheads), presumably corresponding to heterochromatin, interspersed with areas of lower density (white arrowheads), presumably corresponding to euchromatin. The nuclei of cells after the induction of histone H1 RNAi for 48 hours lack the dense chromatin domains. The scale bar represents 500 nm.

Knockdown of histone H1 did not result in obvious changes in the structure or staining intensity of the nucleus as monitored by fluorescence microscopy using DNA staining with DAPI (data not shown). To investigate the effect of histone H1 depletion at the ultrastructural level, we performed transmission electron microscopy (TEM) analysis on parental bloodstream form *T. brucei*, or cells where H1 RNAi had been induced for 48 hours (Fig. 6). The nucleus of *T. brucei* has particularly darkly stained areas (with an electron density comparable to that of the nucleolus) which presumably correspond to heterochromatin. These dark areas are interspersed with more lightly-stained areas which are likely to contain euchromatin [53]. We find that H1 knockdown results in a dramatic loss of the darkly-stained areas (black arrows in Fig. 6), resulting in a homogeneously-stained nucleoplasm. This is consistent with our MNase results showing a general increase in chromatin accessibility after H1 knockdown, indicating that depletion of H1 has an effect on the structure of heterochromatin in *T. brucei*.

### Histone H1 is required for silencing *VSG* ESs in *T. brucei*

**Figure 7:**
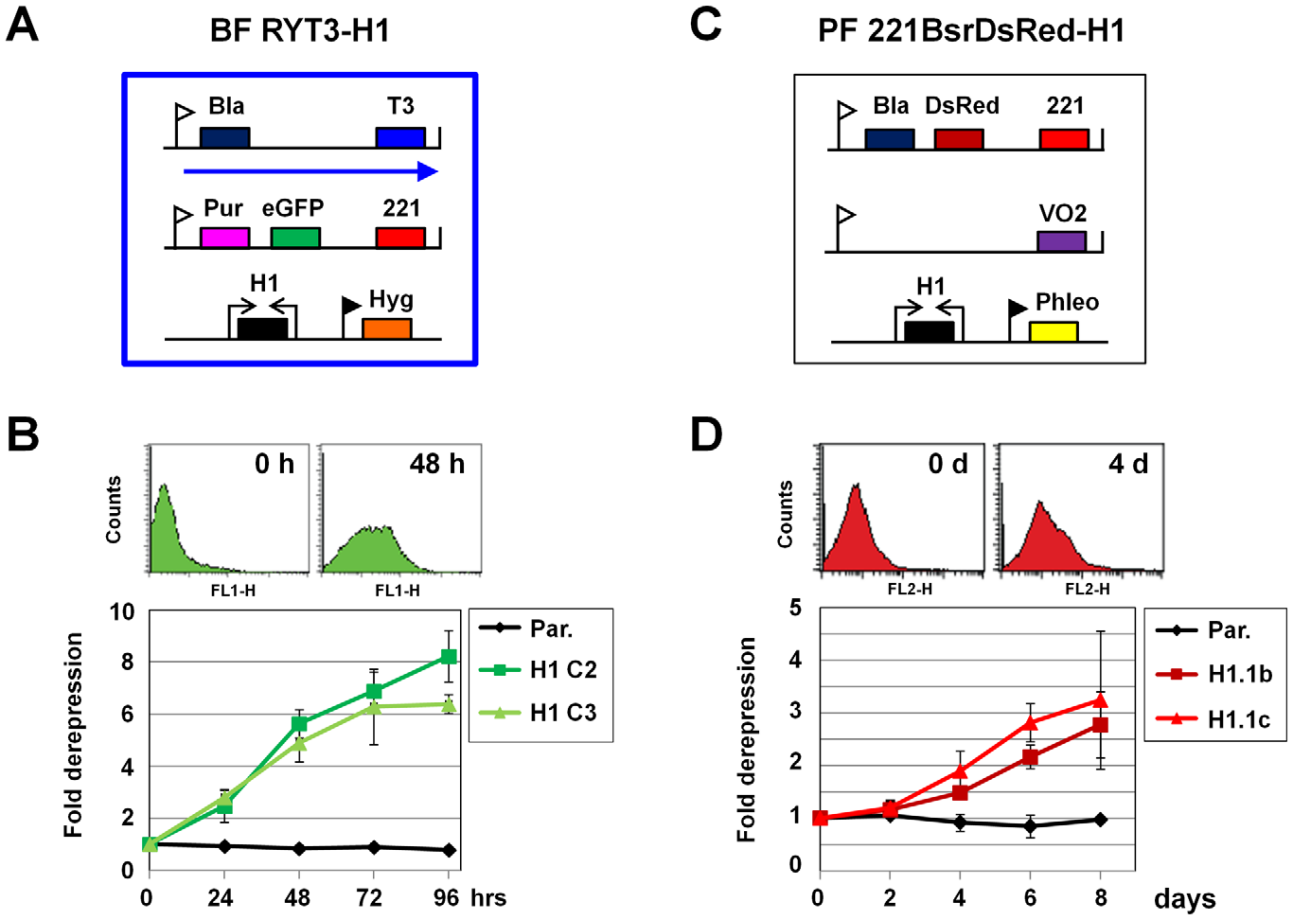
Derepression of silent ESs after knockdown of histone H1. **A.** Schematic of the BF *T. brucei* RYT3-H1 line, which is as described in panel 5A. Histone H1 RNAi can be produced from two opposing tetracycline inducible T7 promoters. Transcription of the active *VSGT3* ES is indicated with an arrow, and derepression of the silent *eGFP* gene located in the inactive *VSG221* ES can be monitored by flow cytometry. **B.** Derepression of the silent *VSG221* ES in BF *T. brucei* RYT3-H1 as measured by flow cytometry. Representative traces from the indicated time points are shown. Fold derepression was calculated by dividing the mean fluorescence value from the induced culture by the mean fluorescence value from a corresponding uninduced culture. The mean from three independent experiments is shown with the standard deviation indicated with error bars. **C.** Schematic of the PF *T. brucei* 221BsrDsRed-H1 cell line used to monitor derepression of a silent *VSG* ES after knockdown of histone H1. **D.** Derepression of a silent ES in PF *T. brucei* after the induction of histone H1 RNAi as monitored using the fluorescent DsRed protein. Representative flow cytometry traces are shown. Fold derepression was calculated as in B for three independent experiments with standard deviation indicated with error bars.

We next investigated if the changes in chromatin structure observed after H1 depletion had functional consequences for transcription. The bloodstream form *T. brucei* RYT3 reporter cell line has *eGFP* immediately downstream of the promoter of the inactive *VSG221* ES, allowing ES derepression to be monitored using GFP fluorescence (Fig. 7A) [42]. Parental *T. brucei* RYT3 and two independent RYT3-H1RNAi clones were grown in the presence or absence of tetracycline and monitored by flow cytometry. Both *T. brucei* RYT3-H1 clones showed 6–8 fold derepression of the silent *VSG221* ES after four days of induction of H1 RNAi (Fig. 7B).

In procyclic form *T. brucei*, all ESs are silenced using a mechanism that appears to be different from that used in bloodstream form *T. brucei* [54]–[59]. Although H1 knockdown in procyclic form *T. brucei* did not result in a detectable change in accessibility of chromatin to endonucleases, we investigated the effect on ES silencing. Our procyclic form *T. brucei* 221BsrDsRed cell line has the *DsRed* reporter gene integrated behind the silent *VSG221* ES promoter (Fig. 7C) [42]. Induction of H1 RNAi led to a modest but reproducible ∼3-fold derepression of the *VSG221* ES promoter in two independent clones, indicating that H1 is required for maximal ES silencing in procyclic form *T. brucei* (Fig. 7D). In contrast to the observed derepression at ESs, steady state levels of actin and γ-tubulin transcripts remained constant after H1 depletion. We also did not observe a significant increase in precursor transcripts derived from the tubulin array following knock-down of H1 (data not shown). However, since *T. brucei* relies on post-transcriptional regulation of its mRNAs, it is possible that any putative minor changes in Pol II transcription following H1 knock-down are absorbed by the cell through mRNA degradation/stability pathways.

### VSG switching in the presence of histone H1 depletion

**Figure 8:**
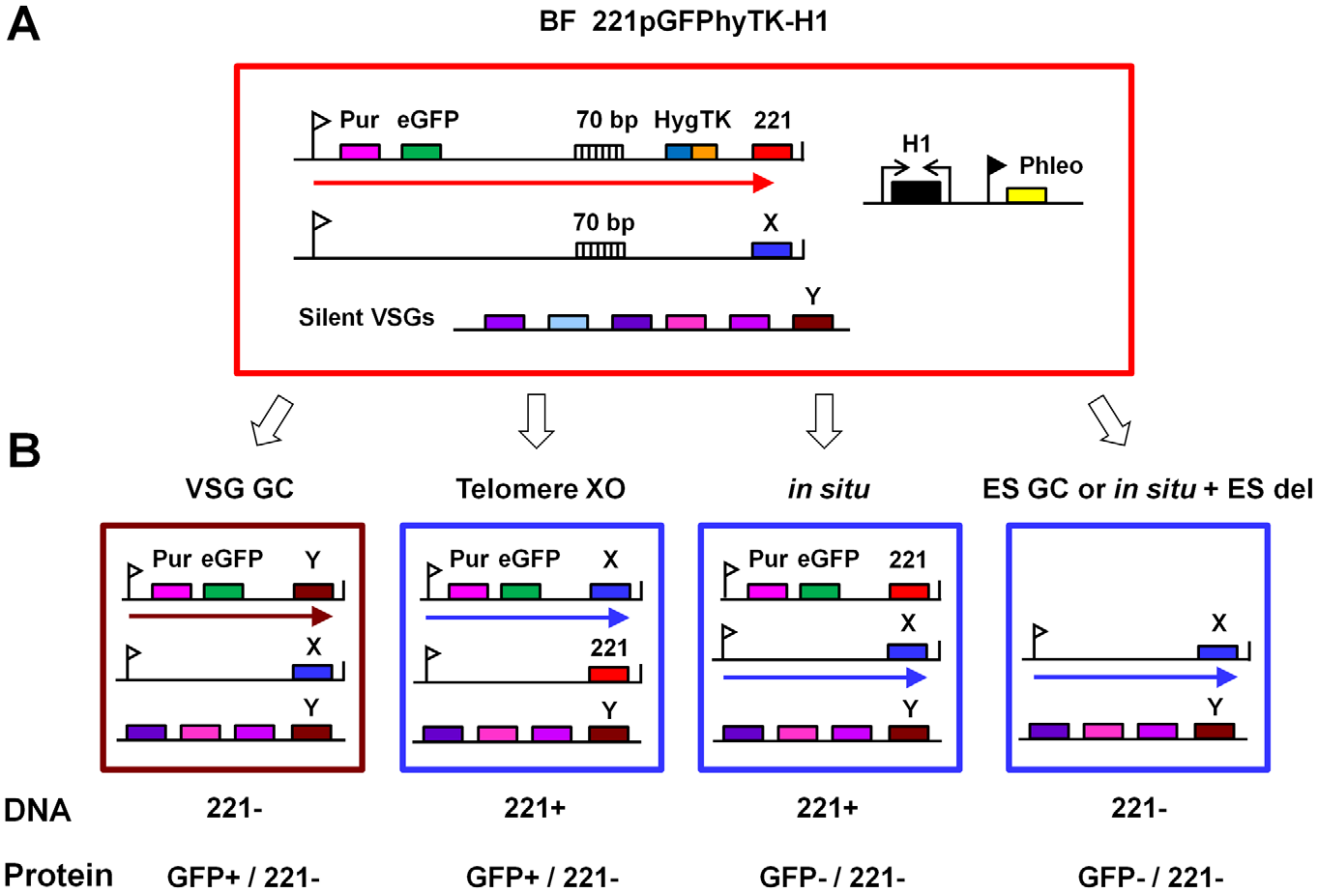
Strategy for determination of VSG switching frequencies after blocking histone H1 synthesis. **A.** Diagram of the BF *T. brucei* 221pGFPhyTK-H1 cell line used for analysis of VSG switching after histone H1 knock-down. The large red box indicates a cell, with a construct containing the puromycin (Pur) resistance gene and *eGFP* integrated immediately behind the active *VSG221* ES promoter (indicated with a flag). A construct containing a fusion protein for hygromycin resistance and thymidine kinase (HYGTK) activity is integrated at the telomeric end of the ES between characteristic 70 bp repeat sequences (70 bp) and the telomeric *VSG221* gene. A silent ES with an unknown *VSG* (X) is indicated below, as well as chromosome internal silent *VSG*s (Y) located in tandem arrays. A construct allowing transcription of histone H1 RNAi (H1) from opposing T7 promoters (arrows) as well as a phleomycin (Phleo) gene transcribed from an rDNA promoter (black flag) has also been introduced. **B.** Schematic of VSG switching mechanisms detectable in our assay, adapted from [66]. Switching the active *VSG* can be mediated through a *VSG* gene conversion (GC), telomere exchange (XO), or *in situ* activation of another ES (*in situ*). In addition, VSG switch events resulting in loss of the *VSG221* ES by either gene conversion (ES GC) or a deletion event after an *in situ* switch (*in situ*+ES del) were identified. Each type of VSG switch event, in addition to mutations in the TK gene itself (not shown), result in resistance to ganciclovir (GCV). Presence of the single copy *VSG221* gene is indicated below (DNA). In addition, expression of the GFP or VSG221 protein are indicated. The schematic is labeled as indicated in panel A.

As histone H1 depletion results in transcriptional derepression of silent ESs, we also investigated the consequences of H1 knockdown on VSG switching frequency. We used a similar strategy to [60], [61], in which a thymidine kinase (TK) gene fused to a drug resistance gene is integrated in the active *VSG221* ES between the 70 bp repeat array and the telomeric *VSG221*. In addition, *eGFP* and a puromycin resistance gene are integrated downstream of the *VSG221* ES promoter (Fig. 8A). *VSG* switch events which cause silencing or loss of the TK gene can be selected for using the nucleoside analogue ganciclovir (GCV). GCV resistance can also arise from mutations in the TK gene [62], however these GCV resistant cells would not have switched their VSG coat as revealed by anti-VSG221 immunofluorescence.

The presence of the ES-located single copy marker genes allows the mechanism of VSG switching to be deduced from the genotype and phenotype of a clone after a VSG switch event (Fig. 8B). After a *VSG* gene conversion (GC), the TK and *VSG221* genes are lost, while the switched cell remains GFP positive. After a switch mediated by a telomere exchange, cells are also GFP positive but retain *VSG221*. A transcriptional (*in situ*) switch results in GFP negative cells without loss of *VSG221*. Finally, a gene conversion can occur which initiates at or near the ES promoter, resulting in the duplication of an entire new ES into the active ES, thereby resulting in its deletion. These switched cells would be GFP negative and would have lost all sequences present in the old *VSG221* ES. Alternatively, as previously observed, the same phenotype can result from an ES *in situ* switch coupled with deletion of the active ES [60], [61], [63], [64].

**Figure 9:**
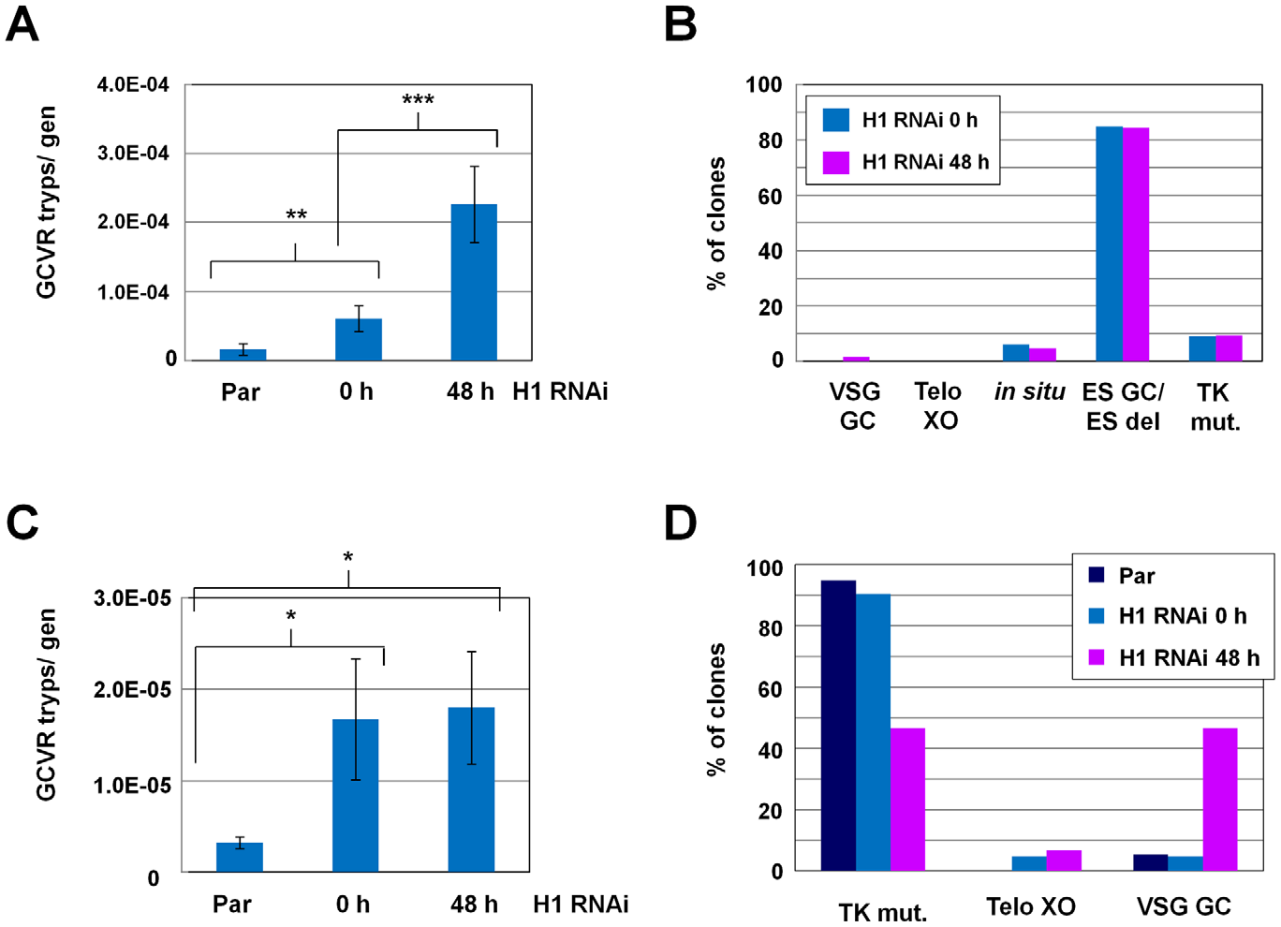
Depletion of histone H1 results in an increase in VSG switching. **A.** The frequency of generation of ganciclovir resistant (GCVR) trypanosomes per generation (GCVR tryps/gen) in parental (Par) *T. brucei* and 221pGFPhyTK-H1 cells in the presence of histone H1 RNAi for the time indicated in hours (h). Bars indicate the mean of independent experiments [parental n = 3; H1 RNAi cells (0 h and 48 h histone H1 RNAi), n = 5]. The standard deviation is indicated with error bars. A higher rate of generation of GCVR clones as a measure for VSG switching was observed in uninduced *T. brucei* 221pGFPhyTK-H1 cells (containing the histone H1 RNAi construct) compared with parental cells (statistical significance **, P<0.01). However, there was a statistically significant increase in this frequency after the induction of histone H1 RNAi for 48 hours (***, P<0.001). **B.** Mechanism of VSG switching in clones from seven independent cultures after the induction of histone H1 RNAi for 48 hours (n = 64) (purple bars) or in four independent cultures generated in the absence of histone H1 RNAi (n = 33) (blue bars). The genotypes and phenotypes of the clones were determined using microscopy and PCR, allowing determination of the VSG switching mechanism used. The percentage of clones that had switched using each mechanism is plotted. VSG switch mechanisms are as indicated in Figure 8, as well as cells which appeared to have mutated the TK gene (TK mut.) as determined by their resistance to GCV and continued expression of VSG221. **C.** Frequency of generation of GCVR clones per generation (freq/gen) in parental (Par) and 221pGFPhyTK-H1 cells grown in the absence (0 h) or presence of histone H1 RNAi for 48 hours (48 h). These experiments were conducted in the presence of puromycin to select for DNA rearrangements in the vicinity of the telomeric *VSG*. Bars indicate the mean of three independent experiments with standard deviation indicated with error bars. As shown in panel A., cells containing the H1 RNAi construct show a statistically significant increase in the frequency of ganciclovir resistant cells (statistical significance *, P = 0.024). This frequency does not further increase after depletion of histone H1 for 48 hours (statistical significance of increase compared with parental *, P = 0.014). **D.** Increased *VSG* switching mediated by gene conversion (GC) in cells depleted for histone H1. VSG switching was monitored in *T. brucei* 221pGFPhyTK-H1 cells in the absence (0 h) or presence of H1 RNAi for 48 hours. VSG switching mechanisms were determined in clones derived from at least three independent cultures for each experiment. All GCVR clones were generated in the presence of puromycin to select for VSG switches mediated by DNA rearrangements at the telomere of the active ES. VSG switching mechanisms were determined using immunofluorescence microscopy and PCR as in panel B. Using parental cells (Par) or uninduced histone H1 RNAi cells (0 h) ∼90% of the obtained clones (Par n = 19; H1 RNAi 0 h n = 21) continued to express VSG221, indicating that they are TK mutants (TK mut.). However, when histone H1 RNAi had been induced for 48 hours, less than half of the generated clones were TK mutants, and ∼33% had switched through VSG gene conversion (VSG GC). Clones that had switched through telomere exchange (Telo XO) were also observed using this assay.

We first established whether H1 knockdown altered the frequency of GCV resistant clones, which can give an indication of changes in VSG switching frequency (Fig. 9A). Cells were removed from drug selection maintaining transcription of the active *VSG221* ES for 48 hours, and H1 RNAi was induced in one culture of TK-expressing cells. Cultures were subsequently serially diluted in 96-well plates in the presence of GCV, and positive wells were scored after 7 days. We observed an average 4-fold increase in the frequency of generation of GCV resistant clones per generation after the induction of a block in H1 synthesis for 48 hours in five independent experiments (Fig. 9A; P = 0.0002). Three cultures of parental cells (without the H1 RNAi construct) were also examined in independent experiments. The frequency of generation of GCV resistant clones in the parental cells was ∼3-fold less than in noninduced cells containing the H1 RNAi construct (P = 0.0086), again indicating leaky production of dsRNA in the absence of tetracycline.

We next determined the mechanism of VSG switching in clonal cell lines where H1 RNAi had been induced for 48 hours (n = 7 independent cultures) or had not been induced (n = 4 independent cultures). Using immunofluorescence microscopy we established if the GCV resistant clones had indeed switched their VSG221 coat, monitored for GFP expression, and determined the presence or absence of the *VSG221* gene by PCR. Data showing validation of this strategy to study VSG switching are in Supplementary Figures S6 and S7. We analysed 33 switched clones derived from uninduced H1 RNAi cells and 64 switched clones from cells where H1 RNAi had been induced for 48 hours. In both cases, we found that the majority of clones had switched by ES gene conversion or an *in situ* switch plus deletion of the *VSG221* ES (Fig. 9B). We also analysed 35 switched clones from four independent cultures derived from an equivalent cell line lacking the histone H1 RNAi construct (parental, data not shown). Here too, the majority of VSG switch events involved deletion of the *VSG221* ES. This high frequency of deletion of the *VSG221* ES after a VSG switch has been previously observed [60], [61], [63], [64].

We still do not know why *VSG* ES deletion events are frequently picked up in *VSG* switching experiments with *T. brucei* 427. VSG switching experiments performed with *T. brucei* have shown variability regarding the predominant VSG switch mechanism used. Some reports have found that *VSG* gene conversion is the most common switching mechanism [60], [65], while others have shown that transcriptional switching between ESs predominates [66], [67]. However, consistent with both these results and those from other laboratories, deletion of the previously active ES frequently occurs [60], [61], [63], [64], [66]. This phenomenon has been frequently observed with the *VSG221* ES, which is often deleted during switch events involving a switch to another ES. The *VSG221* ES is unusually large with extensive duplications and triplications. In addition, it contains unusually short regions of 70 bp repeats which normally facilitate gene conversion [8], [9]. As the *VSG221* ES has been hypothesised to have a particularly low frequency of inactivation, it has been postulated that if the rate of switch off of this ES drops low enough, telomere deletion events resulting in its loss are an alternative way of resolving an unfavourable double-expressor state [64]. This *VSG221* ES deletion event is not a consequence of the TK negative selection system, as these events have also been uncovered in experiments using positive selection for ES activation using drugs or negative selection against *VSG221* using VSG RNAi [66].

We attempted to investigate the effect of H1 depletion on VSG switch events involving DNA rearrangements near the telomeric *VSG*. We repeated our VSG switching assays in the presence of puromycin selection to maintain expression of the *VSG221* ES. This procedure selects for cells that continue to transcribe the active *VSG221* ES, but have silenced (through telomere exchange) or lost (through VSG gene conversion) the HYGTK fusion gene. As with the previous switching assay, this procedure will also select for cells with mutations in the TK gene [60], [61], [63], [64]. Using these conditions we also observed an increase in GCV-resistant clones in cells containing the histone H1 RNAi construct, although this frequency did not further increase after induction of H1 RNAi, presumably as H1 was already significantly depleted in the noninduced cells containing the H1 RNAi construct (Fig. 9C). We analysed the VSG switching mechanisms that had occurred in clonal cell lines derived from at least three independent cultures each of parental cells (n = 19), uninduced *T. brucei* H1 RNAi cells (n = 21), and cells induced for H1 RNAi for 48 h (n = 15). Here we found that our protocol had enriched for cells that continue to express VSG221, and are probably TK mutants (Fig. 9D).

However, we also observed that a higher percentage of the clones in which H1 had been depleted had indeed switched their VSG via gene conversions, indicating that histone H1 in *T. brucei* possibly plays a role in suppressing DNA rearrangements at the telomeric end of the ES. One possibility explaining these data, is that what we observe here are two potentially distinct effects of histone H1 depletion. Leaky H1 RNAi in the uninduced sample results in an increase in the frequency of TK mutants, but does not significantly affect the VSG switching frequency. However, further depletion of histone H1 after tetracycline induction of H1 RNAi causes an increase in VSG switching frequency.

**Figure 10:**
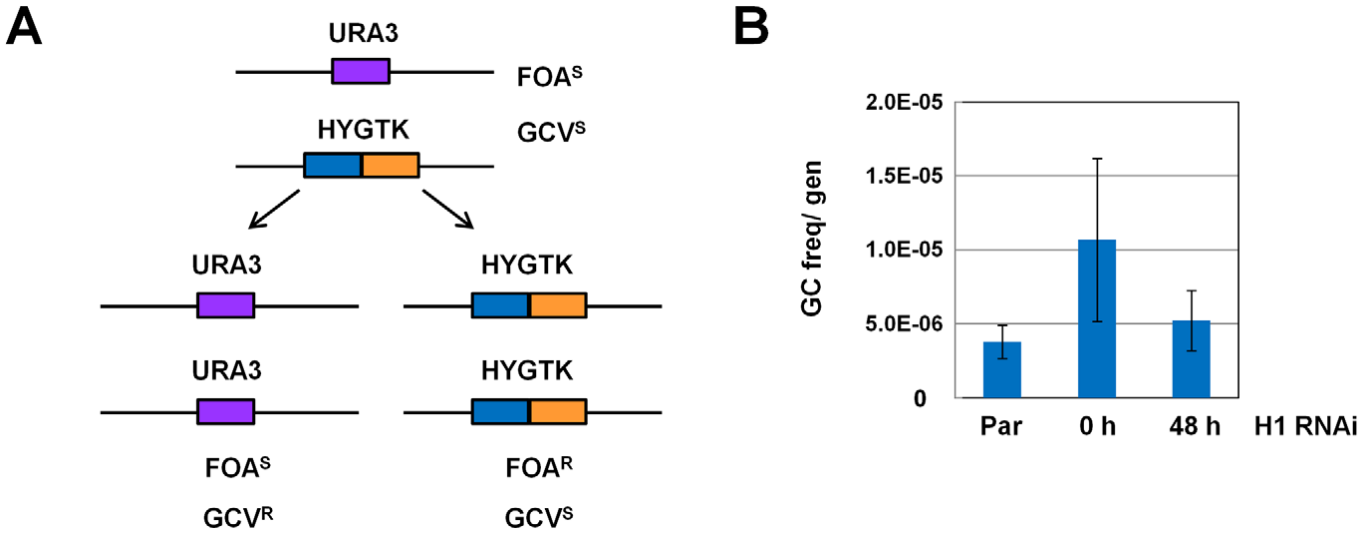
Histone H1 depletion does not affect recombination frequency at the Pol II-transcribed URA3 locus. **A.** The experimental assay for gene conversion is as described in [60], [61]. One allele of the *URA3* gene (violet box) is replaced by a hygromycin resistance/thymidine kinase fusion gene (HYGTK) (blue and orange boxes). Gene conversion at this locus either results in cells which have two copies of the *URA3* gene and are sensitive to FOA (FOA^S^) and resistant to ganciclovir (GCV^R^). Alternatively, the cells have two copies of the HYGTK gene and are FOA resistant (FOA^R^) and sensitive to ganciclovir (GCV^S^). **B.** Frequency of gene conversion at the *URA3* locus in parental (Par) cells or those containing the histone H1 RNAi construct. Cells were removed from hygromycin selection for 48 hours, and one culture of H1 RNAi cells had histone H1 RNAi induced for 48 hours. Cells were then plated in the presence of either GCV or FOA and the number of positive wells were scored after eight days. The value shown is the sum frequency of FOA^R^ and GCV^R^ clones, which can be considered to have undergone gene conversion (GC), divided by the number of generations undergone by each cell line. Bars show the mean of three independent experiments with standard deviation indicated with error bars. The differences between the samples were not statistically significant.

We next investigated if the rate of DNA recombination in the Pol II-transcribed *URA3* locus was also affected by H1 knockdown. We didn't observe dramatic changes in chromatin structure at the *URA3* locus after knockdown of H1, although H1 depletion did result in an increase in MNase accessibility of chromatin at other Pol II transcription units (Fig. 5E). However, rates of gene conversion at loci other than *URA3* are difficult to measure in the absence of negative selectable markers which can select for both alleles. As we could not exclude that minor (and for us undetectable) changes in chromatin structure were nonetheless occurring at the *URA3* locus which could be affecting DNA recombination, we used an assay described in [60], [61] to measure the frequency of gene conversion at this locus after H1 knockdown (Fig. 10A). *T. brucei* expressing URA3 is sensitive to 5-fluoroorotic acid (FOA). We replaced one allele of *URA3* with the hygromycin resistance/thymidine kinase fusion gene (HYGTK) in bloodstream form *T. brucei* that either did (H1 RNAi) or did not (Par) contain the H1 RNAi construct. Removal of cells from hygromycin selection allows gene conversion at the *URA3* locus to occur. This results in cells with either two copies of the URA3 gene (which are sensitive to FOA but resistant to GCV), or two copies of the HYGTK gene (which are sensitive to GCV but resistant to FOA).

Parental and H1 RNAi URA3/HYGTK *T. brucei* cells were expanded for 48 hours in the absence of hygromycin selection. During this period, one of the H1 RNAi cultures was induced with tetracycline (H1 RNAi 48 h). We next placed cells under either FOA or GCV selection, and scored for positive wells after eight days. Validation of a selection of these clones by PCR confirmed that, as expected, GCV resistant clones lacked the *HYGTK* gene while FOA resistant clones lacked the *URA3* gene (data not shown). The frequency of positive wells in both FOA and GCV-containing plates was divided by the number of generation times in the absence of hygromycin to obtain the total frequency of gene conversion per generation for each sample (Fig. 10B). We did not observe a significant difference in frequency of gene conversion at the *URA3* locus in parental (Par) or uninduced cells, compared with cells in which histone H1 had been depleted for 48 hours (n = 3).

## Discussion

We find that the single-domain histone H1 proteins play an essential role in the maintenance of higher order chromatin structure in bloodstream form *T. brucei*. H1 depletion results in a general disruption of electron dense material in the nucleus which appears to correspond to heterochromatin. In addition, H1 knock-down results in an increase in the accessibility of chromatin to MNase in bloodstream *T. brucei*, as well as a marked opening of chromatin structure at the silent ES promoters. In agreement with this, H1 knockdown results in derepression of silent ES promoters, and also appears to lead to an increase in VSG switching. These data all argue that H1 could be one of the layers of control allowing the parasite to tightly control expression of its extensive *VSG* repertoire. H1 knockdown in procyclic form *T. brucei* resulted in only moderate derepression of ESs, and no obvious changes in global chromatin structure as assessed using MNase accessibility. It is possible that the chromatin of wild-type procyclic form *T. brucei* is in a more open state than that found in bloodstream form *T. brucei* [45], or alternatively that H1 interacts with chromatin differently in procyclic form compared with bloodstream form *T. brucei*.

*T. brucei* histone H1 has unusual properties. While H1 proteins normally have a tripartite domain structure including a central globular domain flanked by relatively unstructured N- and C-terminal domains, kinetoplastid H1 proteins have only a single domain rich in lysine, alanine, and proline residues [33]. Here we show that *T. brucei* H1 proteins, while missing domains important for function in other eukaryotes, can still facilitate higher order chromatin structure. It is possible that the mechanism by which single-domain H1 proteins compact chromatin is different from that used by the tripartite H1 proteins [21]. In *T. brucei*, both bloodstream and procyclic form life-cycle stages still grow at reduced rates even if histone H1 is depleted down to very low levels. Histone H1 is nonessential in a number of unicellular eukaryotes [16]–[18]. Although histone H1 depletion in *T. brucei* results in a mild growth phenotype, it is possible that here too these proteins are not essential. However, in the absence of a true genetic H1 knockout (complicated by the fact that there are at least five histone H1 genes) it is not possible for us to establish this point definitively. It is also unclear if the different isoforms of *T. brucei* H1 perform distinct roles or have overlapping functions.

While detailed biochemical analysis of H1 proteins has been performed in *T. brucei*, *T. cruzi*, *L. major*, and most recently *L. braziliensis*, it has not yet been thoroughly investigated how these proteins function *in vivo*. *T. cruzi* has three H1 genes, and phosphorylation sites of the encoded proteins have been mapped [68]. Interestingly, phosphorylated H1 seems to be regulated according to both the cell and developmental cycle of *T. cruzi* [68]–[70]. The phosphorylated H1 also has a distinct localization compared to the non-phosphorylated proteins, and is enriched in the area of the nucleolus [69]. *Leishmania* species have at least two H1 proteins (*L. brasiliensis* has three) [71] that in *L. major* have been shown to be developmentally regulated [72]. Overexpression of H1 in this species affects both chromatin structure and parasite virulence [38], [73]. However, the challenges of working with a multi-gene family as well as the limited availability of tools for genetic manipulation, has precluded loss of function analysis of H1 in these organisms.

In addition to providing insight into the role of H1, this study also provides information on the chromatin structure of *T. brucei*. Genomic areas that are actively transcribed by Pol I including the rDNA, active ESs, and the procyclin loci are highly depleted of nucleosomes, and have an unusually open chromatin structure [13], [14]. We now find that the linker histone H1 is also depleted from these highly transcribed Pol I transcription units. Interestingly, histone H1 as well as histone H3 are also depleted from the procyclin promoter even in bloodstream form *T. brucei*, where procyclin loci are transcriptionally silent. This could indicate the presence of an open chromatin structure associated with ‘paused’ Pol I complexes. Pol I is thought to initiate transcription at procyclin promoters in bloodstream form *T. brucei*, which is not fully processive [74]. In contrast, in Pol II transcription units including actin, γ-tubulin, the *URA3* locus and other single copy genes, the chromatin structure as investigated using histone H1 distribution and MNase accessibility in parental cells appears comparable to that of silent ESs. This indicates that a highly open chromatin structure is not a prerequisite for processive Pol II transcription in *T. brucei*.

It has previously been found that the *T. brucei* nucleus is heterogeneously stained in EM thin sections [6], [53], [75]. The dark and lightly-stained nuclear areas are thought to correspond to heterochromatin and euchromatin respectively, although it is not clear exactly which genomic regions are located in these areas. Here, we show that the darker-stained heterochromatic regions of the nucleus disappear following depletion of H1 in bloodstream form *T. brucei*. Our ChIP experiments show that H1 is enriched at transcriptionally silent regions, including repeat regions and the *VSG* basic copy array, which can be assumed to be present in the form of heterochromatin. As our MNase accessibility experiments indicate that H1 knockdown causes the chromatin in these areas to become more open, it is therefore highly likely that the darkly stained regions of the *T. brucei* nucleus visualized by EM are in fact areas of more tightly packaged DNA. It is possible that H1 knockdown also has an effect on other aspects of subnuclear organization, including telomere clustering and the distribution of minichromosomes, thereby leading to some of the functional consequences we observe. Interestingly, knock-down of NUP-1, a lamin-like protein important for nuclear structure in *T. brucei*, has also been shown to affect VSG switching frequency [76]. In addition, another aspect of nuclear organization, the association of sister chromatids during mitosis, has been proposed to play a role in regulation of antigenic variation, as depletion of cohesin subunits results in an increased VSG switching frequency [77].

What is the role of H1 mediated chromatin structure in an organism that has little transcriptional control? Although H1 knock-down resulted in an increase in accessibility of chromatin to nucleases in bloodstream form *T. brucei*, this effect was not observed in procyclic form *T. brucei.* It is clear that one function of H1 could be to facilitate different processes required for efficient antigenic variation in *T. brucei*. We also find that histone H1 plays a role in silencing inactive ESs particularly in bloodstream form *T. brucei*. In contrast, depletion of H1 in *S. cerevisiae* had no effect on telomeric silencing [17]. Our results argue that the higher-order chromatin structure maintained by histone H1 in *T. brucei* is critical for complete ES silencing in bloodstream form *T. brucei*. In addition to being enriched on silent *VSG* ESs, H1 in *T. brucei* also appears to play a role in maintenance of the closed chromatin state found at the silent basic copy *VSG*s. We find that H1 is enriched on silent *VSG*s including the *VSG118* basic copy gene, and H1 knock-down results in a more open chromatin structure at this locus.

For antigenic variation to be effective, rates of DNA recombination need to be suppressed to levels that prevent unnecessarily rapid depletion of the *VSG* repertoire. VSG switching rates of the laboratory adapted *T. brucei* 427 strain are significantly lower than in strains which are closer to field isolates [65]. The factors that contribute to these high VSG switch rates in some isolates have not yet been determined. However, we feel that the first step is to identify proteins which modulate the frequency of VSG switching in experimentally amenable laboratory strains, before verifying their relevance in the field.

We find that depletion of H1 not only disrupts *VSG* ES silencing, but also appears to result in an increase in the frequency of VSG switching. It is possible that depletion of H1 from the silent ESs causes an increase in homologous recombination. This is consistent with a proposed role for H1 in suppressing recombination events and maintaining genome stability in yeast [27], [78]. Homologous recombination plays a major role in VSG switching [2], [79]. Depletion of BRCA2, Rad51 and RAD51-related proteins all decrease *VSG* switching frequency, presumably through disruption of homologous recombination pathways [80]–[83]. In contrast, knockout of subunits of the *T. brucei* RTR complex, which is thought to resolve recombination intermediates, causes an increase in the *VSG* switching frequency [60], [61]. Possibly higher-order chromatin structures maintained by H1 could suppress the formation of recombination intermediates between the highly similar *VSG* ESs, as has been found in *S. cerevisiae* in the rDNA transcription units [27], [78].

Depletion of H1 had a much more dramatic effect on chromatin structure in the region immediately downstream of the silent ES promoters compared with at the telomeric *VSG* gene. It is possible that in the absence of H1 other factors (such as RAP1) are still affecting chromatin structure in the region adjacent to the telomere [84]. Depletion of H1 also affected the chromatin structure of a *VSG* located in a basic copy array.

Although our ChIP and MNase accessibility data indicate that H1 could affect chromatin structure in at least some Pol II transcription units, knockdown of H1 did not result in increased MNase accessibility at the *URA3* locus. Since H1 knockdown does not alter chromatin structure at the *URA3* locus, it is perhaps unsurprising that rates of homologous recombination at this locus do not increase after H1 depletion. Although there was some effect of H1 depletion on the chromatin of other Pol II loci including actin or γ-tubulin, H1 depletion had a less dramatic effect on the chromatin structure at these loci compared with at the ES promoter regions. In *S. cerevisiae*, histone H1 has been shown to suppress homologous recombination at the repetitive Pol I transcribed rDNA loci, but had no effect on homologous recombination at another locus outside of the rDNA [78].

Here, we show that H1 containing heterochromatin is not only involved in silencing *VSG* ES promoters, but could also be involved in suppressing VSG switching. Our studies on *T. brucei* histone H1 provide insights into the unique structure and role of chromatin in these parasites, and these data therefore provide additional evidence for the key role played by chromatin structure and remodeling in the processes involved in antigenic variation in these important human pathogens.

## Materials and Methods

### Trypanosome strains and culture

Bloodstream form (BF) *T. brucei brucei* strain 427 was cultured in HMI-9 medium [85] supplemented with foetal calf serum (FCS) and the appropriate drugs at 37°C, 5% CO_2_. During the course of these experiments the amount of FCS in the HMI-9 was reduced from 20% to 15%, with no observable effect on cell growth or the kinetics of the histone H1 RNAi phenotype. Procyclic form (PF) *T. brucei* was cultured in SDM-79 medium supplemented with 10% FCS, 5 µg/ml hemin, and the appropriate drugs at 27°C. Cell densities were determined using a haemocytometer.

The BF *T. brucei* RYT3-H1 cell line was generated by transfection of the p2T7-H1hy histone H1 RNAi construct into the *T. brucei* RYT3 cell line. This parental cell line is based on the BF *T. brucei* ‘single-marker’ [86] cell line, and contains a blasticidin resistance gene in the active *VSGT3* ES, and a 221GP1 puromycin/eGFP construct integrated behind the silent *VSG221* ES promoter [42], [87]. This BF *T. brucei* RYT3-H1 cell line was used for experiments monitoring the general phenotype after the induction of histone H1 RNAi including growth, protein and transcript levels, micrococcal nuclease (MNase) sensitivity, and global nuclear architecture as investigated using electron microscopy (EM).

For the experiments analysing VSG switching, BF *T. brucei* ‘single marker’ cells were transfected with the 221GP1 construct (containing the puromycin and *eGFP* genes) which integrates immediately downstream of the active *VSG221* ES promoter [87]. Subsequently, the p2T7-H1ph histone H1 RNAi construct (described below) was introduced and selected for using the phleomycin resistance gene. Finally, a construct containing a hygromycin resistance-thymidine kinase fusion gene (HYGTK) [60](see below) was integrated into the active *VSG221* ES between the 70 bp repeat sequences and the *VSG221* gene itself.

To examine the effect of histone H1 depletion in procyclic form *T. brucei*, the PF *T. brucei* DsRed-H1 cell line was created. To do this, the p2T7-H1ph construct was transfected into the 221BsrDsRed parental *T. brucei* line [42], which is based on the *T. brucei* 29-13 line [86], and contains a blasticidin resistance gene and a DsRed fluorescent protein gene in the silent *VSG221* ES. Phleomycin selection was used to select for stable integration of the construct, and clonal cell lines were obtained. ChIP experiments were performed using the BF *T. brucei* HNI 221+ [64] and PF *T. brucei* 221BsrDsRed cell lines [42].

### DNA constructs

Five genes have been annotated as histone H1-like in the *T. brucei brucei* genome with the TriTrypDB accession numbers: Tb11.42.0005, Tb11.42.0006, Tb11.42.0007, Tb11.55.0001, and Tb11.39.0008. Protein sequence alignments were performed using ClustalW. To produce double-stranded RNA (RNAi) homologous to the different polymorphic members of the histone H1 gene family in *T. brucei*, two histone H1 fragments (∼600 bp each) corresponding to different histone H1 genes were PCR amplified from the *T. b. brucei* 427 genome, and cloned in tandem into the p2T7-177 RNAi vector which targets *T. brucei* minichromosomes [51]. The first histone H1 fragment includes the coding region of Tb11.42.0005 as well as the intergenic region between Tb11.42.0005 and Tb11.42.0006, and was amplified using primers HisH1Con1_183s 5′-TATGGATTCAGACAACTGCTGTCCCCAAG-3′ (BamHI link) and HisH1Con1_884as 5′-TATAAGCTTGAGCAGCAGATGCCTTCG-3′ (HindIII link). The second histone H1 fragment includes the intergenic region between Tb11.39.0008 and the coding region of Tb11.55.0001, and was amplified using primers HisH1Con2_524s 5′-TATAAGCTTCCCGCTATTAGACACGCTATG-3′ (HindIII link) and HisH1Con2_1173as 5′-TATCTCGAGGATGCGCTCACGCCTTCT-3′ (XhoI link). These two histone H1 fragments were inserted by triple ligation into the p2T7-177-Hygro or p2T7-177-Phleo constructs to generate the p2T7-H1hy and p2T7-H1ph constructs. These contained a ∼1.2 kb fragment producing dsRNA capable of RNAi-mediated knockdown of all five predicted *T. brucei* histone H1 genes.

In order to integrate a construct containing the HYGTK fusion gene into the *VSG221* ES, we employed a strategy similar to that of Kim and Cross, 2009. First, we cut out a ∼2.5 kb region of 70 bp repeat sequence from the construct RM3173 [67] and cloned it into pBluescript. We next amplified by PCR a region of the *VSG221* ES between the 70 bp repeats and the *VSG221* gene using primers VSG221TAR_55928s 5′-TATGGATCCGACGAATACAAACCATAAATAAATGC-3′ (BamHI link) and VSG221TAR_56433as 5′-TATGCGGCCGCCAAGACGTGGTGCAATCATC-3′ (NotI link) and cloned it into the same vector. We finally inserted the HYGTK fusion gene (a generous gift of Nina Papavasiliou and George Cross) flanked by an upstream tubulin intergenic region containing an RNA splice site and a downstream actin intergenic region containing a polyadenylation signal in between the 70 bp repeat fragment and the *VSG221* ES targeting fragment. The vector was cut with *Hind*III and *Not*I prior to transfection, and correct integration confirmed by PCR linking.

### Protein and nucleic acid analysis

To create antibodies specific for *T. brucei* histone H1 proteins, two histone H1 peptides were designed, synthesized, and injected into rabbits (Eurogentec). The *T. brucei* histone H1 peptide sequences are: N-KKVAPKKVAGKKAAA-C (amino acids 59–73 of Tb11.55.0001) and N-AAPKKAVAKKAAPKK-C (amino acids 6–20 of Tb11.55.0001). Affinity purification of antibodies specific for these peptides was carried out by Eurogentec. The specificity of the *T. brucei* histone H1 antibodies was confirmed using Western blots comparing lysates from wild-type *T. brucei* with lysates from *T. brucei* after RNAi-mediated knockdown of histone H1. To create antibodies specific for VSG221, a ∼500 bp fragment was amplified by PCR using primers VSG221Ab_243s 5′-GCAAGTATATACGCTGAAATAAATCAC-3′ and VSG221Ab_741as 5′-TGTTTGGCTGTTCGCTACTGTGAC-3′ and cloned into the pRSETA vector (Invitrogen) for expression of an N-terminally tagged 6×-His-tagged protein. Protein was purified under denaturing conditions and was used for immunisation of rabbits (Eurogentec).

Tris-Tricine SDS-PAGE electrophoresis was performed with 16% polyacrylamide gels before being transferred to PVDF membrane (GE Healthcare) using standard protocols. Blots were probed with affinity-purified anti-histone H1 peptide antibodies, anti-histone H3 antibody (ab1791, AbCam), anti-La antibodies (a gift from Elisabetta Ullu), or anti-BiP (a gift from Jay Bangs). Detection of the appropriate peroxidase-coupled secondary antibody was performed using ECL Plus (Amersham) detection kit.

To determine the affinity of histone H1 for chromatin in *T. brucei* strain (BF) HNI 221+ or (PF) 221BsrDsRed, 1×10^7^ cells per sample were centrifuged at 1000 rcf for 7 min. Cell pellets were washed twice and the supernatants removed. For “total” samples, cell pellets were resuspended in 200 µl hot Tricine SDS-PAGE sample buffer. For other samples, cell pellets were resuspended in PBS+1% Triton X-100 containing either 0, 100, 200, 400, or 800 mM NaCl. Roche protease inhibitors (−EDTA) were also included in each lysis solution. Lysates were incubated on ice for 20 min, followed by centrifugation at 16000 rcf for 20 min at 4°C. 25 µl of each supernatant was removed to a fresh tube. Hot 2× Tricine SDS-PAGE buffer (25 µl) was then added to each supernatant and samples were boiled for 5 min. The rest of the supernatant was discarded from each sample, and the pellets resuspended in 200 µl hot Tricine SDS-PAGE sample buffer. All other protein lysates were prepared by resuspension of a washed cell pellet in hot Tricine sample buffer to a final concentration of 10^5^ cell equivalents per µl.

RNA isolation, cDNA production and qPCR analysis of steady state transcript levels was performed as described in Narayanan *et al* 2011 [43].

### Perchloric acid extraction of histone H1 proteins

*T. brucei* PF 221BsrDsRed cells (1×10^9^) were centrifuged at 1000 rcf for 10 min and washed twice with 10 ml trypanosome wash solution (100 mM NaCl, 3 mM MgCl_2_, 20 mM Tris-HCl pH 7.5 at 4°C [88]. The cells were then washed once in 10 ml transcription buffer (150 mM sucrose, 20 mM L-glutamic acid, 20 mM HEPES-KOH pH 7.7, 3 mM MgCl_2_, 1 mM DTT, Roche complete protease inhibitors −EDTA, and Sigma phosphatase inhibitor cocktails 2 and 3), at 4°C [88]. The cell pellet was resuspended in 300 µl transcription buffer and kept on ice for 20 min. Cells were lysed by douncing using short burst of rapid strokes for 45 minutes on ice using a Wheaton 2 ml dounce as modified from [88]. Lysis (>80%) was confirmed by light microscopy, then cells were centrifuged at 16000 rcf for 10 min at 4°C. After removal of the supernatant, the pellet was resuspended in 200 µl 5% perchloric acid, and incubated on ice for 40 min [89].

The sample was then centrifuged at 16000 rcf for 20 min at 4°C, and the supernatant transferred to fresh tubes (100 µl/1.5 ml tube). Proteins were precipitated using acetone, and dried pellets were resuspended in Laemmli SDS-PAGE sample buffer. Samples were boiled for 5 min before loading on Tris-glycine SDS-PAGE gels for analysis using standard protocols.

### Immunofluorescence microscopy

For localization of histone H1 proteins, immunofluorescence microscopy was performed essentially as described [43]. *T. brucei* BF HNI(221+) and PF 221BsrDsRed cells were used. Histone H1 was detected using the affinity purified *T. brucei* anti-histone H1 peptide antibody and an Alexa-594-conjugated anti-rabbit secondary antibody (Invitrogen). The *T. brucei* nucleolus was detected using the monoclonal L1C6 antibody (Devaux et al 2007)(a gift from Keith Gull) and an anti-mouse Alexa-488-conjugated secondary antibody (Invitrogen). Slides were visualized on a Zeiss Axio Imager.M1 microscope equipped with a Zeiss AxioCam MRm camera using AxioVision Rel 4.8 software. Images were cropped and brightness and contrast uniformly adjusted using Adobe Photoshop.

### Chromatin immunoprecipitation

Chromatin immunoprecipitation (ChIP) experiments were performed as described [14]. Histone H3 was immunoprecipitated using an anti-H3 antibody (ab1791, AbCam) and served as a positive control for the ChIP procedure. A cross-linked sample in which no antibody had been added served as a negative control. For ChIP of histone H1 proteins, 20 µl of affinity-purified histone H1 antibody was used, with 20 µl of pre-immune serum from the same rabbit used as a negative control. Analysis of the ChIP material was performed using either quantitative PCR (qPCR) using the primer sets described in [14] or by slot-blotting and hybridization with radio-labelled probes as described [14]. All qPCR reactions were performed with Agilent Technologies Brilliant II SybrGreen qPCR master mix with low ROX on an Applied Biosystems 7500 Fast Real-Time PCR system. All statistical analyses are unpaired, two-tailed t-tests with a 95% confidence interval, and were performed using GraphPad Prism software.

### Micrococcal nuclease treatment and sucrose gradient fractionation

Micrococcal nuclease (MNase) treatment of chromatin was performed essentially as described [14]. For small-scale preparations, 2×10^7^ cells per sample were permeabilized with digitonin and treated with either 0.0625, 0.125, 0.25, or 0.5 units of MNase (Worthington Biochemicals). Isolated DNA was then loaded on a gel, and bands quantitated using ImageJ. For MNase treatment followed by sucrose gradient fractionation, 2–3×10^8^ cells per sample were permeabilized and treated with 2.5 units MNase per 1×10^8^ cells. Solubilised chromatin was then loaded onto a 5–30% discontinuous sucrose gradient, and the different nucleosome species were fractionated by ultracentrifugation at 40,000 rpm (Beckman SW41 Ti rotor) for 16 h at 4°C. Fractions were treated with proteinase K and RNase A, and DNA isolated by phenol-chloroform extraction followed by ethanol precipitation. After visualization of some of the isolated DNA on a gel, the remaining DNA fractions were pooled into mono-, di-, di/tri-, tri-tetra-, and >tetra nucleosomal fractions. The amount of each DNA target present in each fraction was determined by qPCR using primer sets described in [14]. The summed amount of each target detected in all fractions was designated as the “total” amount, and the amount detected in the mononucleosome fraction was plotted as a percent of that total.

### Transmission electron microscopy

Cells were fixed in 2.5% glutaraldehyde, post-fixed in 1% osmium tetroxide and stained with 2% uranyl acetate before dehydration through a series of different ethanol concentrations and embedding in Agar 100 epoxy resin. Thin sections (90 nm) were stained with lead citrate and examined using an FEI Tecnai 12 transmission electron microscope at 80 kV according to [90].

### Flow cytometry

Derepression of the silent *VSG221* ES in the *T. brucei* BF RYT3 and PF 221BsrDsRed cell lines was monitored by flow cytometry as described [40], [42], [43] with minor modifications. Briefly, histone H1 RNAi was induced with the addition of 1 µg/ml tetracycline. At each time point, ∼1×10^6^ cells were centrifuged, washed, and fixed in 2% paraformaldehyde for one hour at room temperature. Fixed cells were then washed and analysed by flow cytometry using a Becton-Dickinson FACSCalibur and CellQuest software (BD). For each time point, fold derepression was calculated by dividing the mean FL1-H or FL2-H fluorescence value of induced cells by the mean fluorescence value of the uninduced cells.

### VSG switching experiments and analysis of switch variants

To determine VSG switching frequencies and mechanisms, we employed a strategy similar to that used in [60], [61] with some modifications. We created the *T. brucei* 221pGFPhyTK cell line, which contains a construct containing a puromycin and *eGFP* gene immediately downstream of the promoter in the active *VSG221* ES, and a construct containing the HYGTK fusion protein gene downstream of the 70 bp repeats in the same ES. The *T. brucei* 221pGFPhyTKH1 cell line contains the same marker genes as 221pGFPhyTK with the addition of the p2T7-H1ph RNAi construct. We maintained both *T. brucei* 221pGFPhyTK (parental) and *T. brucei* 221pGFPhyTKH1 cells under puromycin and hygromycin selection to select for a homogenous population expressing the *VSG221* ES. We subsequently removed these cells from drug selection and allowed them to switch for 48 hours. Tetracycline (1 µg/ml) was added to an additional culture of *T. brucei* 221pGFPhyTKH1 cells to induce RNAi of histone H1, also for 48 hours. Cells were then plated in 3-fold serial dilutions in the presence of 4 µg/ml ganciclovir (GCV) (Sigma), and VSG switching frequencies were calculated based on the number of positive wells (containing growing cells), the number of cells plated for each dilution, and the number of generations undergone in the absence of drugs. We have determined the plating efficiency of the different cell lines using different experimental conditions (i.e. in the presence or absence of various drugs) and did not find a clear difference in plating efficiency (data not shown). We therefore did not normalise our data for this variable. However as the *T. brucei* lines capable of histone H1 RNAi have a longer generation time than parental cells, we expressed our data as switch/recombination events per generation. However, the effect of H1 depletion is also apparent even when the data is not normalised for number of generation times.

Individual switched clones were analysed for GFP fluorescence, VSG221 expression (using immunofluorescence microscopy), and PCR for the *VSG221* gene (primers VSG221_243s 5′-GCAAGTATATACGCTGAAATAAATCAC-3′ and VSG221_741as 5′- TGTTTGGCTGTTCGCTACTGTGAC-3′). PCR amplification of the large subunit of RNA polymerase I was performed as a positive control (Pol1_4102s 5′-CTGGATCCAGCGCCGTTCCACGCGAGA-3′ and Pol1_4554as 5′-GACTCGAGCTATCCCCAATCCGTGCCGTCCCG-3′). Pulsed-field gels were performed essentially as described [63]. The gel was stained and processed for blotting onto Hybond XL membrane (GE Healthcare) and probed for *VSG221* and *VSG1.8* using standard protocols. The probes were amplified by PCR using VSG221_121s 5′- TGCCAGGTCTCCGAG-3′, VSG221_1056as 5′-GCTGCTCGGATATGAGCTTTT-3′, VSG1.8_179s 5′-CAATCTCGAGGCTCACAAAAGTCTG-3′, and VSG1.8_1022as 5′-GCTGGGATCCTAGCCTCGAAAAATG-3′.

### URA3 gene conversion assay

Determination of the frequency of gene conversion at the *T. brucei* URA3 locus was performed as described [60], [61]. Briefly, the pyrFEKO-HYG construct (a gift from G. Cross) containing targeting fragments for the URA3 gene (Tb927.5.3810) flanking a hygromycin resistance/thymidine kinase fusion gene was transfected into both BF *T. brucei* 221pGFP cells or BF *T. brucei* 221pGFPH1 cells (containing the histone H1 RNAi construct). Correct integration of the construct was confirmed by PCR amplification using primers URA3uplink_9228s (5′-AGAAAGAACCGTACCGCAGA-3′) and Hygro_125as (5′-CCTACATCGAAGCTGAAAGCAC-3′) for upstream linking and TKlink_2370s (5′-TTTACGGGCTACTTGCCAAT-3′) and URA3dnlink_12374as (5′-AGGGGGAAACAGCGTAAGTT-3′) for downstream linking. Cells were grown in the absence of hygromycin selection for 48 hours. Cells were then plated (5×10^5^ cells per sample) in the presence of either 6 µg/ml 5-Flouroorotic acid (Sigma) or 30 µg/ml GCV. Plates were scored 8 days later, and the gene conversion frequency was calculated by dividing the frequency of positive wells for each selection method by the number of doubling times undergone by each culture in the absence of hygromycin.

## Supporting Information

Figure S1 **Association of histone H1 proteins with chromatin in procyclic form (PF)** ***T. brucei*** **.** *T. brucei* 221BsrDsRed cells were lysed with 1% Triton X-100 in the presence of increasing concentrations of NaCl (molarity indicated above the lanes). Total indicates total lysate. Pellet and supernatant (Sup.) fractions were analysed by Western blotting with antibodies against histone H1, histone H3 or the RNA binding protein La. The size markers in kiloDaltons (kDa) are indicated on the left.
(TIF)

Figure S2 **Genomic distribution of histone H1 in procyclic form** ***T. brucei*** **.** **A.** Schematic of the procyclic form (PF) *T. brucei* PF 221BsrDsRed cell line used for ChIP experiments indicated as a large box containing two relevant ESs. The blasticidin (Bla) resistance gene and the *DsRed* gene are inserted immediately behind the promoter (white flag) of the *VSG221* ES. The *VSGVO2* ES is shown below. **B.** Representative slot blots showing the association of histone H1 and histone H3 proteins with 50 bp repeat sequences flanking ESs, or 177 bp repeat sequences which comprise the *T. brucei* minichromosomes. Experiments were performed with no antibody (No ab) or pre-immune serum (Pre-imm.) as negative controls. For each sample, 10% of the ChIP material was loaded on a slot blot and compared with 0.1% of the total input. **C.** Quantitation of material immunoprecipitated (% IP) using anti-histone H3 (H3) or anti-histone H1 (H1) in the slot blots shown in panel B. Bars show the mean of three experiments with standard deviation indicated with error bars. Two negative controls were used, no antibody (No ab) or pre-immune serum (pre-im) from the rabbit used to produce the histone H1 antibody. **D.** Distribution of histone H1 within the genome of procyclic form *T. brucei* as determined using qPCR analysis of immunoprecipitated material. The bars indicate the amount precipitated (% IP) using the anti-histone H1 antibody (H1) or the pre-immune serum (Pre-imm.) with the standard deviation from three experiments indicated with error bars. Statistically significant amounts of histone H1 (P<0.05) were found at all loci. The regions analysed include the actin, γ-tubulin (γ-tub) and spliced leader (SL) gene loci. The SL intergenic region (int.), promoter region (pro.), or the SL gene itself (SL) are indicated. The ribosomal DNA (rDNA) regions analysed include the rDNA intergenic region (int.), promoter (pro.) or the 18S rDNA gene (18S). The EP procyclin locus analysed includes the region upstream of the EP promoter (up.), the promoter (pro.), or the EP procyclin gene (EP). A higher level of histone H1 was immunoprecipitated upstream of the rDNA promoter compared with at the promoter region itself, with the statistical significance indicated with asterisks (** indicates P<0.01). ES sequences analysed include a region immediately upstream of the ES promoter (up.) as well as the ES promoter itself (pro.) with these primer pairs expected to recognise most if not all ESs. Sequences analysed within the *VSG221* ES include the blasticidin resistance gene (Blast) and the telomeric *VSG221*. *VSG118* is located in the silent chromosome internal *VSG* arrays.
(TIF)

Figure S3 **Statistically significant chromatin immunoprecipitation (ChIP) using anti-histone H1 antibody compared with pre-immune serum.** Different *T. brucei* genomic regions are indicated in the table. ChIP was performed in both bloodstream form and procyclic form *T. brucei*. The P values are shown for the ChIP performed using anti-histone H1 anti-serum compared with the pre-immune serum, with the significance of the results indicated with asterisks.
(TIF)

Figure S4 **Histone H1 depletion in procyclic** ***T. brucei*** **does not result in an increase in open chromatin.** Parental procyclic form (PF) *T. brucei* 221BsrDsRed (Par) or cells in which histone H1 had been depleted using RNAi for four days (H1+4 d) were permeabilized, and the chromatin digested with increasing amounts of micrococcal nuclease (MNase). Lanes contain material digested with different MNase concentrations (Lane 1 = 0.0625 units MNase, lane 2 = 0.125 units, lane 3 = 0.25 units, lane 4 = 0.5 units). Purified DNA was subsequently analysed on agarose gels stained with ethidium bromide. DNA corresponding to mono-, di- and tri-nucleosome species are indicated, along with undigested DNA. A representative gel is shown.
(TIF)

Figure S5 **Depletion of histone H1 has a minimal effect on steady-state transcript levels derived from the γ-tubulin, EP procyclin and 18S rRNA gene loci.** RNA was isolated from indicated time points in hours (h) following induction of histone H1 RNAi, and used as templates for cDNA production. Quantitative PCR (qPCR) was then performed. Relative levels of each transcript were determined, first after normalization to actin, and next in comparison with the level of each transcript at the 0 hour timepoint. Error bars indicate the standard deviation from three independent experiments.
(TIF)

Figure S6 **Confirmation of VSG switching mechanisms through genotype analysis.** **A.** The presence of the single copy *VSG221* gene was determined using PCR. Representative PCR reactions were analysed on 1% agarose gels with the gene encoding the RNA polymerase I (Pol I) large subunit used as a positive control. Genomic DNA from single-marker BF *T. brucei* cells was analysed as a positive control for the presence of the *VSG221* gene (lane 1). DNA from a cell line which has deleted the *VSG221* ES as a result of a switch event was used as a negative control (lane 2). DNA from clones which have undergone an *in situ* switch is shown in lanes 7, 11 and 17. DNA from a clone which has switched as a result of a gene conversion is shown in lane 13. DNA from clones which have switched as a consequence of an ES gene conversion or deletion event is shown in lanes 2–4, 6, 8–10, 14–16 and 18. DNA from clones which have not switched their VSG, but contain a mutation in the TK gene is shown in lanes 5, 12 and 19. **B.** Pulsed field gel electrophoresis (PFGE) analysis confirms the presence or absence of the *VSG221* gene in a representative selection of switched clones as determined using PCR in panel A. An ethidium bromide (EtBr) stained PFGE gel of *T. brucei* chromosomes is shown with a DNA marker (L) indicated in megabases (Mb) on the left. The gel was subsequently blotted and probed for *VSG221* or *VSG1.8*. DNA from the unswitched parental control is shown in lane 1 compared with various clones which have switched their VSG. DNA from clones which have undergone an ES gene conversion or deletion (ES GC/ES del) (lanes 2–5), an *in situ* switch (lanes 6–8) or a *VSG* gene conversion (VSG GC) (lane 9) are shown in comparison. The blots were probed for both *VSG1.8* and *VSG221*, which appear to be located on the same chromosome. This allows us to monitor the *VSG221* containing chromosome, even in clones where the *VSG221* gene has been deleted. This can reveal changes in chromosomal size resulting from deletions of large regions of the chromosome after *VSG* switching (consistent with the results of [63]). In several clones (lanes 3–5), the *VSG221* ES deletion event is indeed significant enough to alter migration of this chromosome. The unshifted *VSG1.8* band that remains in clones 3–5 is most likely due to hybridisation of the probe with a *VSG1.8* gene on a chromosome that remains in the compression zone. Panels A and B show the analysis of different sets of switched clones.
(TIF)

Figure S7 **Analysis of VSG switching using fluorescence microscopy.** Fixed *T. brucei* was subjected to immunofluorescence using an anti-VSG221 antibody. Cells were also monitored for GFP fluorescence, and DNA stained with DAPI. A differential interference contrast (DIC) image is shown for reference. For each channel, a similar exposure time was used to image the different clones. Scale bar is 10 µm.
(TIF)

## References

[1] Taylor JE, Rudenko G (2006) Switching trypanosome coats: what's in the wardrobe? Trends Genet 22: 614–620. 16908087 10.1016/j.tig.2006.08.003

[2] Horn D, McCulloch R (2010) Molecular mechanisms underlying the control of antigenic variation in African trypanosomes. Curr Opin Microbiol 13: 700–705. 20884281 10.1016/j.mib.2010.08.009 PMC3117991

[3] Berriman M, Ghedin E, Hertz-Fowler C, Blandin G, Renauld H, et al (2005) The genome of the African trypanosome Trypanosoma brucei. Science 309: 416–422. 16020726 10.1126/science.1112642

[4] Marcello L, Barry JD (2007) Analysis of the VSG gene silent archive in Trypanosoma brucei reveals that mosaic gene expression is prominent in antigenic variation and is favored by archive substructure. Genome Res 17: 1344–1352. 17652423 10.1101/gr.6421207 PMC1950903

[5] Hutchinson OC, Picozzi K, Jones NG, Mott H, Sharma R, et al (2007) Variant Surface Glycoprotein gene repertoires in Trypanosoma brucei have diverged to become strain-specific. BMC Genomics 8: 234. 17629915 10.1186/1471-2164-8-234 PMC1934917

[6] Ersfeld K (2011) Nuclear architecture, genome and chromatin organisation in Trypanosoma brucei. Res Microbiol 162: 626–636. 21392575 10.1016/j.resmic.2011.01.014

[7] Rudenko G (2010) Epigenetics and transcriptional control in African trypanosomes. Essays Biochem 48: 201–219. 20822495 10.1042/bse0480201

[8] Berriman M, Hall N, Sheader K, Bringaud F, Tiwari B, et al (2002) The architecture of variant surface glycoprotein gene expression sites in Trypanosoma brucei. Mol Biochem Parasitol 122: 131–140. 12106867 10.1016/s0166-6851(02)00092-0

[9] Hertz-Fowler C, Figueiredo LM, Quail MA, Becker M, Jackson A, et al (2008) Telomeric expression sites are highly conserved in Trypanosoma brucei. PLoS One 3: e3527. 18953401 10.1371/journal.pone.0003527 PMC2567434

[10] Gunzl A, Bruderer T, Laufer G, Schimanski B, Tu LC, et al (2003) RNA polymerase I transcribes procyclin genes and variant surface glycoprotein gene expression sites in Trypanosoma brucei. Eukaryot Cell 2: 542–551. 12796299 10.1128/EC.2.3.542-551.2003 PMC161450

[11] Kolev NG, Franklin JB, Carmi S, Shi H, Michaeli S, et al (2010) The transcriptome of the human pathogen Trypanosoma brucei at single-nucleotide resolution. PLoS Pathog 6: e1001090. 20838601 10.1371/journal.ppat.1001090 PMC2936537

[12] Figueiredo LM, Cross GA, Janzen CJ (2009) Epigenetic regulation in African trypanosomes: a new kid on the block. Nat Rev Microbiol 7: 504–513. 19528957 10.1038/nrmicro2149

[13] Figueiredo LM, Cross GA (2010) Nucleosomes are depleted at the VSG expression site transcribed by RNA polymerase I in African trypanosomes. Eukaryot Cell 9: 148–154. 19915072 10.1128/EC.00282-09 PMC2805297

[14] Stanne TM, Rudenko G (2010) Active VSG expression sites in Trypanosoma brucei are depleted of nucleosomes. Eukaryot Cell 9: 136–147. 19915073 10.1128/EC.00281-09 PMC2805301

[15] Thoma F, Koller T (1977) Influence of histone H1 on chromatin structure. Cell 12: 101–107. 561660 10.1016/0092-8674(77)90188-x

[16] Shen X, Yu L, Weir JW, Gorovsky MA (1995) Linker histones are not essential and affect chromatin condensation in vivo. Cell 82: 47–56. 7606784 10.1016/0092-8674(95)90051-9

[17] Escher D, Schaffner W (1997) Gene activation at a distance and telomeric silencing are not affected by yeast histone H1. Mol Gen Genet 256: 456–461. 9393443 10.1007/s004380050589

[18] Patterton HG, Landel CC, Landsman D, Peterson CL, Simpson RT (1998) The biochemical and phenotypic characterization of Hho1p, the putative linker histone H1 of Saccharomyces cerevisiae. J Biol Chem 273: 7268–7276. 9516420 10.1074/jbc.273.13.7268

[19] Laybourn PJ, Kadonaga JT (1991) Role of nucleosomal cores and histone H1 in regulation of transcription by RNA polymerase II. Science 254: 238–245. 1718039 10.1126/science.254.5029.238

[20] Juan LJ, Utley RT, Vignali M, Bohm L, Workman JL (1997) H1-mediated repression of transcription factor binding to a stably positioned nucleosome. J Biol Chem 272: 3635–3640. 9013616 10.1074/jbc.272.6.3635

[21] Harvey AC, Downs JA (2004) What functions do linker histones provide? Mol Microbiol 53: 771–775. 15255891 10.1111/j.1365-2958.2004.04195.x

[22] Trollope AF, Sapojnikova N, Thorne AW, Crane-Robinson C, Myers FA (2010) Linker histone subtypes are not generalized gene repressors. Biochim Biophys Acta 1799: 642–652. 20800709 10.1016/j.bbagrm.2010.08.007

[23] Woodcock CL, Skoultchi AI, Fan Y (2006) Role of linker histone in chromatin structure and function: H1 stoichiometry and nucleosome repeat length. Chromosome Res 14: 17–25. 16506093 10.1007/s10577-005-1024-3

[24] Hellauer K, Sirard E, Turcotte B (2001) Decreased expression of specific genes in yeast cells lacking histone H1. J Biol Chem 276: 13587–13592. 11278859 10.1074/jbc.M011196200

[25] Shen X, Gorovsky MA (1996) Linker histone H1 regulates specific gene expression but not global transcription in vivo. Cell 86: 475–483. 8756729 10.1016/s0092-8674(00)80120-8

[26] Fan Y, Nikitina T, Zhao J, Fleury TJ, Bhattacharyya R, et al (2005) Histone H1 depletion in mammals alters global chromatin structure but causes specific changes in gene regulation. Cell 123: 1199–1212. 16377562 10.1016/j.cell.2005.10.028

[27] Downs JA, Kosmidou E, Morgan A, Jackson SP (2003) Suppression of homologous recombination by the Saccharomyces cerevisiae linker histone. Mol Cell 11: 1685–1692. 12820979 10.1016/s1097-2765(03)00197-7

[28] Alsford S, Horn D (2004) Trypanosomatid histones. Mol Microbiol 53: 365–372. 15228519 10.1111/j.1365-2958.2004.04151.x

[29] Mandava V, Fernandez JP, Deng H, Janzen CJ, Hake SB, et al (2007) Histone modifications in Trypanosoma brucei. Mol Biochem Parasitol 156: 41–50. 17714803 10.1016/j.molbiopara.2007.07.005 PMC2012948

[30] Strahl BD, Allis CD (2000) The language of covalent histone modifications. Nature 403: 41–45. 10638745 10.1038/47412

[31] Hecker H, Betschart B, Bender K, Burri M, Schlimme W (1994) The chromatin of trypanosomes. Int J Parasitol 24: 809–819. 7982743 10.1016/0020-7519(94)90007-8

[32] Zhou YB, Gerchman SE, Ramakrishnan V, Travers A, Muyldermans S (1998) Position and orientation of the globular domain of linker histone H5 on the nucleosome. Nature 395: 402–405. 9759733 10.1038/26521

[33] Kasinsky HE, Lewis JD, Dacks JB, Ausio J (2001) Origin of H1 linker histones. FASEB J 15: 34–42. 11149891 10.1096/fj.00-0237rev

[34] Allan J, Mitchell T, Harborne N, Bohm L, Crane-Robinson C (1986) Roles of H1 domains in determining higher order chromatin structure and H1 location. J Mol Biol 187: 591–601. 3458926 10.1016/0022-2836(86)90337-2

[35] Caterino TL, Hayes JJ (2011) Structure of the H1 C-terminal domain and function in chromatin condensation. Biochem Cell Biol 89: 35–44. 21326361 10.1139/O10-024 PMC3787537

[36] Misteli T, Gunjan A, Hock R, Bustin M, Brown DT (2000) Dynamic binding of histone H1 to chromatin in living cells. Nature 408: 877–881. 11130729 10.1038/35048610

[37] Burri M, Schlimme W, Betschart B, Kampfer U, Schaller J, et al (1993) Biochemical and functional characterization of histone H1-like proteins in procyclic Trypanosoma brucei brucei. Parasitol Res 79: 649–659. 8295902 10.1007/BF00932507

[38] Masina S, Zangger H, Rivier D, Fasel N (2007) Histone H1 regulates chromatin condensation in Leishmania parasites. Exp Parasitol 116: 83–87. 17207482 10.1016/j.exppara.2006.11.002

[39] Zu T, Goyard S, Rappuoli R, Scarlato V (1996) DNA binding of the Bordetella pertussis H1 homolog alters in vitro DNA flexibility. J Bacteriol 178: 2982–2985. 8631692 10.1128/jb.178.10.2982-2985.1996 PMC178039

[40] Denninger V, Fullbrook A, Bessat M, Ersfeld K, Rudenko G (2010) The FACT subunit TbSpt16 is involved in cell cycle specific control of VSG expression sites in Trypanosoma brucei. Mol Microbiol 78: 459–474. 20879999 10.1111/j.1365-2958.2010.07350.x PMC3034197

[41] Figueiredo LM, Janzen CJ, Cross GA (2008) A histone methyltransferase modulates antigenic variation in African trypanosomes. PLoS Biol 6: e161. 18597556 10.1371/journal.pbio.0060161 PMC2443197

[42] Hughes K, Wand M, Foulston L, Young R, Harley K, et al (2007) A novel ISWI is involved in VSG expression site downregulation in African trypanosomes. Embo J 26: 2400–2410. 17431399 10.1038/sj.emboj.7601678 PMC1864976

[43] Narayanan MS, Kushwaha M, Ersfeld K, Fullbrook A, Stanne TM, et al (2011) NLP is a novel transcription regulator involved in VSG expression site control in Trypanosoma brucei. Nucleic Acids Res 39: 2018–2031. 21076155 10.1093/nar/gkq950 PMC3064810

[44] Gruter E, Betschart B (2001) Isolation, characterisation and organisation of histone H1 genes in African trypanosomes. Parasitol Res 87: 977–984. 11763441 10.1007/s004360100483

[45] Schlimme W, Burri M, Bender K, Betschart B, Hecker H (1993) Trypanosoma brucei brucei: differences in the nuclear chromatin of bloodstream forms and procyclic culture forms. Parasitology 107 (Pt 3) 237–247. 8233587 10.1017/s003118200007921x

[46] Happel N, Doenecke D (2009) Histone H1 and its isoforms: contribution to chromatin structure and function. Gene 431: 1–12. 19059319 10.1016/j.gene.2008.11.003

[47] Arhin GK, Shen S, Perez IF, Tschudi C, Ullu E (2005) Downregulation of the essential Trypanosoma brucei La protein affects accumulation of elongator methionyl-tRNA. Mol Biochem Parasitol 144: 104–108. 16055205 10.1016/j.molbiopara.2005.06.006

[48] Devaux S, Kelly S, Lecordier L, Wickstead B, Perez-Morga D, et al (2007) Diversification of function by different isoforms of conventionally shared RNA polymerase subunits. Mol Biol Cell 18: 1293–1301. 17267688 10.1091/mbc.E06-09-0841 PMC1838988

[49] Zomerdijk JC, Ouellette M, ten Asbroek AL, Kieft R, Bommer AM, et al (1990) The promoter for a variant surface glycoprotein gene expression site in Trypanosoma brucei. EMBO J 9: 2791–2801. 1697265 10.1002/j.1460-2075.1990.tb07467.x PMC551989

[50] Wickstead B, Ersfeld K, Gull K (2004) The small chromosomes of Trypanosoma brucei involved in antigenic variation are constructed around repetitive palindromes. Genome Res 14: 1014–1024. 15173109 10.1101/gr.2227704 PMC419779

[51] Wickstead B, Ersfeld K, Gull K (2002) Targeting of a tetracycline-inducible expression system to the transcriptionally silent minichromosomes of Trypanosoma brucei. Mol Biochem Parasitol 125: 211–216. 12467990 10.1016/s0166-6851(02)00238-4

[52] Alibu VP, Storm L, Haile S, Clayton C, Horn D (2005) A doubly inducible system for RNA interference and rapid RNAi plasmid construction in Trypanosoma brucei. Mol Biochem Parasitol 139: 75–82. 15610821 10.1016/j.molbiopara.2004.10.002

[53] Ogbadoyi E, Ersfeld K, Robinson D, Sherwin T, Gull K (2000) Architecture of the Trypanosoma brucei nucleus during interphase and mitosis. Chromosoma 108: 501–513. 10794572 10.1007/s004120050402

[54] Navarro M, Cross GA (1998) In situ analysis of a variant surface glycoprotein expression-site promoter region in Trypanosoma brucei. Mol Biochem Parasitol 94: 53–66. 9719510 10.1016/s0166-6851(98)00049-8

[55] Navarro M, Cross GA, Wirtz E (1999) Trypanosoma brucei variant surface glycoprotein regulation involves coupled activation/inactivation and chromatin remodeling of expression sites. Embo J 18: 2265–2272. 10205179 10.1093/emboj/18.8.2265 PMC1171309

[56] Rudenko G, Blundell PA, Taylor MC, Kieft R, Borst P (1994) VSG gene expression site control in insect form Trypanosoma brucei. Embo J 13: 5470–5482. 7957113 10.1002/j.1460-2075.1994.tb06882.x PMC395505

[57] Rudenko G, Blundell PA, Dirks-Mulder A, Kieft R, Borst P (1995) A ribosomal DNA promoter replacing the promoter of a telomeric VSG gene expression site can be efficiently switched on and off in T. brucei. Cell 83: 547–553. 7585957 10.1016/0092-8674(95)90094-2

[58] Horn D, Cross GA (1997) Position-dependent and promoter-specific regulation of gene expression in Trypanosoma brucei. Embo J 16: 7422–7431. 9405371 10.1093/emboj/16.24.7422 PMC1170342

[59] Horn D, Cross GA (1995) A developmentally regulated position effect at a telomeric locus in Trypanosoma brucei. Cell 83: 555–561. 7585958 10.1016/0092-8674(95)90095-0

[60] Kim HS, Cross GA (2010) TOPO3alpha influences antigenic variation by monitoring expression-site-associated VSG switching in Trypanosoma brucei. PLoS Pathog 6: e1000992. 20628569 10.1371/journal.ppat.1000992 PMC2900300

[61] Kim HS, Cross GA (2011) Identification of Trypanosoma brucei RMI1/BLAP75 homologue and its roles in antigenic variation. PLoS One 6: e25313. 21980422 10.1371/journal.pone.0025313 PMC3182221

[62] Valdes J, Taylor MC, Cross MA, Ligtenberg MJ, Rudenko G, et al (1996) The viral thymidine kinase gene as a tool for the study of mutagenesis in Trypanosoma brucei. Nucleic Acids Res 24: 1809–1815. 8657559 10.1093/nar/24.10.1809 PMC145877

[63] Cross M, Taylor MC, Borst P (1998) Frequent loss of the active site during variant surface glycoprotein expression site switching in vitro in Trypanosoma brucei. Mol Cell Biol 18: 198–205. 9418867 10.1128/mcb.18.1.198 PMC121476

[64] Rudenko G, Chaves I, Dirks-Mulder A, Borst P (1998) Selection for activation of a new variant surface glycoprotein gene expression site in Trypanosoma brucei can result in deletion of the old one. Mol Biochem Parasitol 95: 97–109. 9763292 10.1016/s0166-6851(98)00099-1

[65] Robinson NP, Burman N, Melville SE, Barry JD (1999) Predominance of Duplicative VSG Gene Conversion in Antigenic Variation in African Trypanosomes. Mol Cell Biol 19: 5839–5846. 10454531 10.1128/mcb.19.9.5839 PMC84433

[66] Aitcheson N, Talbot S, Shapiro J, Hughes K, Adkin C, et al (2005) VSG switching in Trypanosoma brucei: antigenic variation analysed using RNAi in the absence of immune selection. Mol Microbiol 57: 1608–1622. 16135228 10.1111/j.1365-2958.2005.04795.x PMC1618954

[67] McCulloch R, Rudenko G, Borst P (1997) Gene conversions mediating antigenic variation in Trypanosoma brucei can occur in variant surface glycoprotein expression sites lacking 70-base-pair repeat sequences. Mol Cell Biol 17: 833–843. 9001237 10.1128/mcb.17.2.833 PMC231809

[68] da Cunha JP, Nakayasu ES, Elias MC, Pimenta DC, Tellez-Inon MT, et al (2005) Trypanosoma cruzi histone H1 is phosphorylated in a typical cyclin dependent kinase site accordingly to the cell cycle. Mol Biochem Parasitol 140: 75–86. 15694489 10.1016/j.molbiopara.2004.12.007

[69] Gutiyama LM, da Cunha JP, Schenkman S (2008) Histone H1 of Trypanosoma cruzi is concentrated in the nucleolus region and disperses upon phosphorylation during progression to mitosis. Eukaryot Cell 7: 560–568. 18281601 10.1128/EC.00460-07 PMC2292618

[70] Marques Porto R, Amino R, Elias MC, Faria M, Schenkman S (2002) Histone H1 is phosphorylated in non-replicating and infective forms of Trypanosoma cruzi. Mol Biochem Parasitol 119: 265–271. 11814578 10.1016/s0166-6851(01)00430-3

[71] Carmelo E, Gonzalez G, Cruz T, Osuna A, Hernandez M, et al (2011) Characterization of monomeric DNA-binding protein Histone H1 in Leishmania braziliensis. Parasitology 138: 1093–1101. 21767437 10.1017/S0031182011000898

[72] Noll TM, Desponds C, Belli SI, Glaser TA, Fasel NJ (1997) Histone H1 expression varies during the Leishmania major life cycle. Mol Biochem Parasitol 84: 215–227. 9084041 10.1016/s0166-6851(96)02801-0

[73] Smirlis D, Bisti SN, Xingi E, Konidou G, Thiakaki M, et al (2006) Leishmania histone H1 overexpression delays parasite cell-cycle progression, parasite differentiation and reduces Leishmania infectivity in vivo. Mol Microbiol 60: 1457–1473. 16796681 10.1111/j.1365-2958.2006.05205.x

[74] Vanhamme L, Berberof M, Le Ray D, Pays E (1995) Stimuli of differentiation regulate RNA elongation in the transcription units for the major stage-specific antigens of Trypanosoma brucei. NucleicAcidsRes 23: 1862–1869. 10.1093/nar/23.11.1862 PMC306955 7596810

[75] Daniels JP, Gull K, Wickstead B (2010) Cell biology of the trypanosome genome. Microbiol Mol Biol Rev 74: 552–569. 21119017 10.1128/MMBR.00024-10 PMC3008170

[76] DuBois KN, Alsford S, Holden JM, Buisson J, Swiderski M, et al (2012) NUP-1 Is a large coiled-coil nucleoskeletal protein in trypanosomes with lamin-like functions. PLoS Biol 10: e1001287. 22479148 10.1371/journal.pbio.1001287 PMC3313915

[77] Landeira D, Bart JM, Van Tyne D, Navarro M (2009) Cohesin regulates VSG monoallelic expression in trypanosomes. J Cell Biol 186: 243–254. 19635842 10.1083/jcb.200902119 PMC2717648

[78] Li C, Mueller JE, Elfline M, Bryk M (2008) Linker histone H1 represses recombination at the ribosomal DNA locus in the budding yeast Saccharomyces cerevisiae. Mol Microbiol 67: 906–919. 18179596 10.1111/j.1365-2958.2007.06101.x

[79] Vink C, Rudenko G, Seifert HS (2011) Microbial antigenic variation mediated by homologous DNA recombination. FEMS Microbiol Rev E-pub ahead of print doi: 10.1111/j.1574-6976.2011.00321.x 10.1111/j.1574-6976.2011.00321.x PMC3334452 22212019

[80] Dobson R, Stockdale C, Lapsley C, Wilkes J, McCulloch R (2011) Interactions among Trypanosoma brucei RAD51 paralogues in DNA repair and antigenic variation. Mol Microbiol 81: 434–456. 21615552 10.1111/j.1365-2958.2011.07703.x PMC3170485

[81] Hartley CL, McCulloch R (2008) Trypanosoma brucei BRCA2 acts in antigenic variation and has undergone a recent expansion in BRC repeat number that is important during homologous recombination. Mol Microbiol 68: 1237–1251. 18430140 10.1111/j.1365-2958.2008.06230.x PMC2408642

[82] McCulloch R, Barry JD (1999) A role for RAD51 and homologous recombination in Trypanosoma brucei antigenic variation. Genes Dev 13: 2875–2888. 10557214 10.1101/gad.13.21.2875 PMC317127

[83] Proudfoot C, McCulloch R (2005) Distinct roles for two RAD51-related genes in Trypanosoma brucei antigenic variation. Nucleic Acids Res 33: 6906–6919. 16326865 10.1093/nar/gki996 PMC1301600

[84] Yang X, Figueiredo LM, Espinal A, Okubo E, Li B (2009) RAP1 is essential for silencing telomeric variant surface glycoprotein genes in Trypanosoma brucei. Cell 137: 99–109. 19345190 10.1016/j.cell.2009.01.037 PMC2673096

[85] Hirumi H, Hirumi K (1989) Continuous cultivation of Trypanosoma brucei blood stream forms in a medium containing a low concentration of serum protein without feeder cell layers. J Parasitol 75: 985–989. 2614608

[86] Wirtz E, Leal S, Ochatt C, Cross GA (1999) A tightly regulated inducible expression system for conditional gene knock-outs and dominant-negative genetics in Trypanosoma brucei. Mol Biochem Parasitol 99: 89–101. 10215027 10.1016/s0166-6851(99)00002-x

[87] Sheader K, te Vruchte D, Rudenko G (2004) Bloodstream form-specific up-regulation of silent vsg expression sites and procyclin in Trypanosoma brucei after inhibition of DNA synthesis or DNA damage. J Biol Chem 279: 13363–13374. 14726511 10.1074/jbc.M312307200

[88] Laufer G, Schaaf G, Bollgonn S, Gunzl A (1999) In vitro analysis of alpha-amanitin-resistant transcription from the rRNA, procyclic acidic repetitive protein, and variant surface glycoprotein gene promoters in Trypanosoma brucei. Mol Cell Biol 19: 5466–5473. 10409736 10.1128/mcb.19.8.5466 PMC84388

[89] Mackeen MM, Kramer HB, Chang KH, Coleman ML, Hopkinson RJ, et al (2010) Small-molecule-based inhibition of histone demethylation in cells assessed by quantitative mass spectrometry. J Proteome Res 9: 4082–4092. 20583823 10.1021/pr100269b PMC4681095

[90] Hoog JL, Gluenz E, Vaughan S, Gull K (2010) Ultrastructural investigation methods for Trypanosoma brucei. Methods Cell Biol 96: 175–196. 20869523 10.1016/S0091-679X(10)96008-1

