# Supplementary figures and images for: Histone H1 Plays a Role in Heterochromatin Formation and VSG Expression Site Silencing in Trypanosoma brucei
Source: PLoS Pathog. 2012 Nov 1;8(11):e1003010. doi: 10.1371/journal.ppat.1003010 (PMC3486875; doi:10.1371/journal.ppat.1003010)

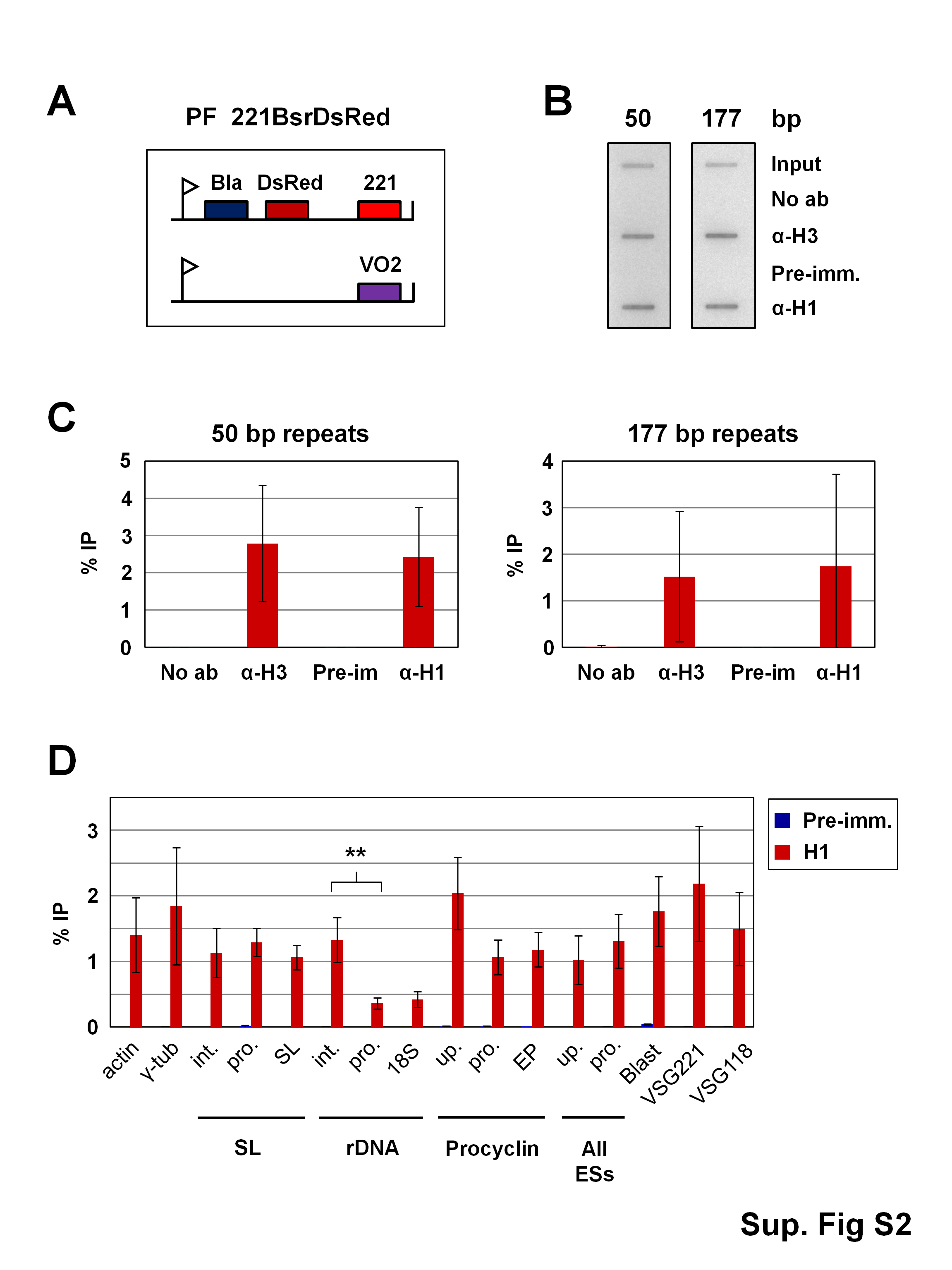

Supplement: Figure S2 — Genomic distribution of histone H1 in procyclic form T. brucei . A. Schematic of the procyclic form (PF) T. brucei PF 221BsrDsRed cell line used for ChIP experiments indicated as a large box containing two relevant ESs. The blasticidin (Bla) resistance gene and the DsRed gene are inserted immediately behind the promoter (white flag) of the VSG221 ES. The VSGVO2 ES is shown below. B. Representative slot blots showing the association of histone H1 and histone H3 proteins with 50 bp repeat sequences flanking ESs, or 177 bp repeat sequences which comprise the T. brucei minichromosomes. Experiments were performed with no antibody (No ab) or pre-immune serum (Pre-imm.) as negative controls. For each sample, 10% of the ChIP material was loaded on a slot blot and compared with 0.1% of the total input. C. Quantitation of material immunoprecipitated (% IP) using anti-histone H3 (H3) or anti-histone H1 (H1) in the slot blots shown in panel B. Bars show the mean of three experiments with standard deviation indicated with error bars. Two negative controls were used, no antibody (No ab) or pre-immune serum (pre-im) from the rabbit used to produce the histone H1 antibody. D. Distribution of histone H1 within the genome of procyclic form T. brucei as determined using qPCR analysis of immunoprecipitated material. The bars indicate the amount precipitated (% IP) using the anti-histone H1 antibody (H1) or the pre-immune serum (Pre-imm.) with the standard deviation from three experiments indicated with error bars. Statistically significant amounts of histone H1 (P<0.05) were found at all loci. The regions analysed include the actin, γ-tubulin (γ-tub) and spliced leader (SL) gene loci. The SL intergenic region (int.), promoter region (pro.), or the SL gene itself (SL) are indicated. The ribosomal DNA (rDNA) regions analysed include the rDNA intergenic region (int.), promoter (pro.) or the 18S rDNA gene (18S). The EP procyclin locus analysed includes the region upstream of the EP promote [file ppat.1003010.s002.tif]

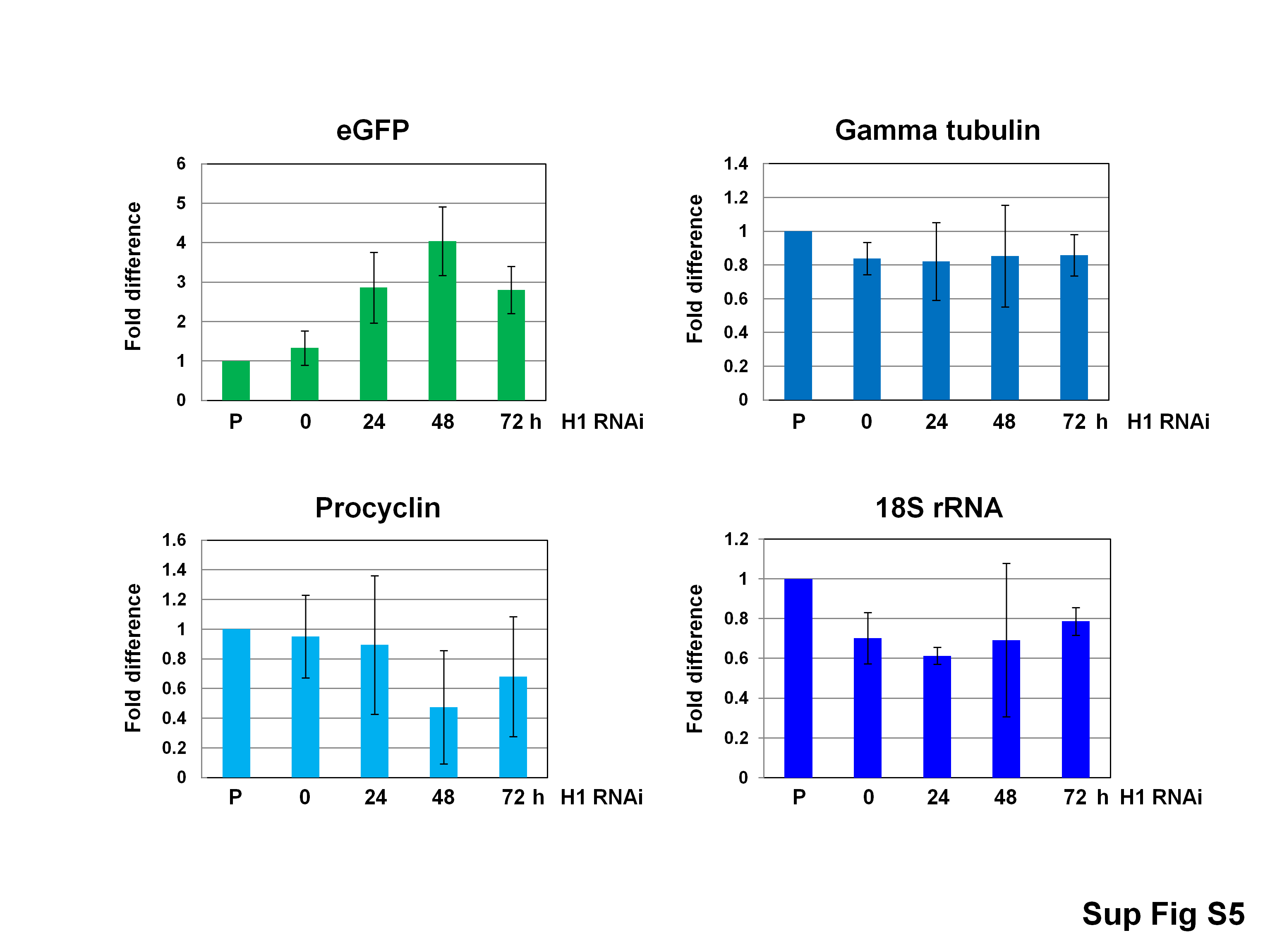

Supplement: Figure S5 — Depletion of histone H1 has a minimal effect on steady-state transcript levels derived from the γ-tubulin, EP procyclin and 18S rRNA gene loci. RNA was isolated from indicated time points in hours (h) following induction of histone H1 RNAi, and used as templates for cDNA production. Quantitative PCR (qPCR) was then performed. Relative levels of each transcript were determined, first after normalization to actin, and next in comparison with the level of each transcript at the 0 hour timepoint. Error bars indicate the standard deviation from three independent experiments. (TIF) [file ppat.1003010.s005.tif]

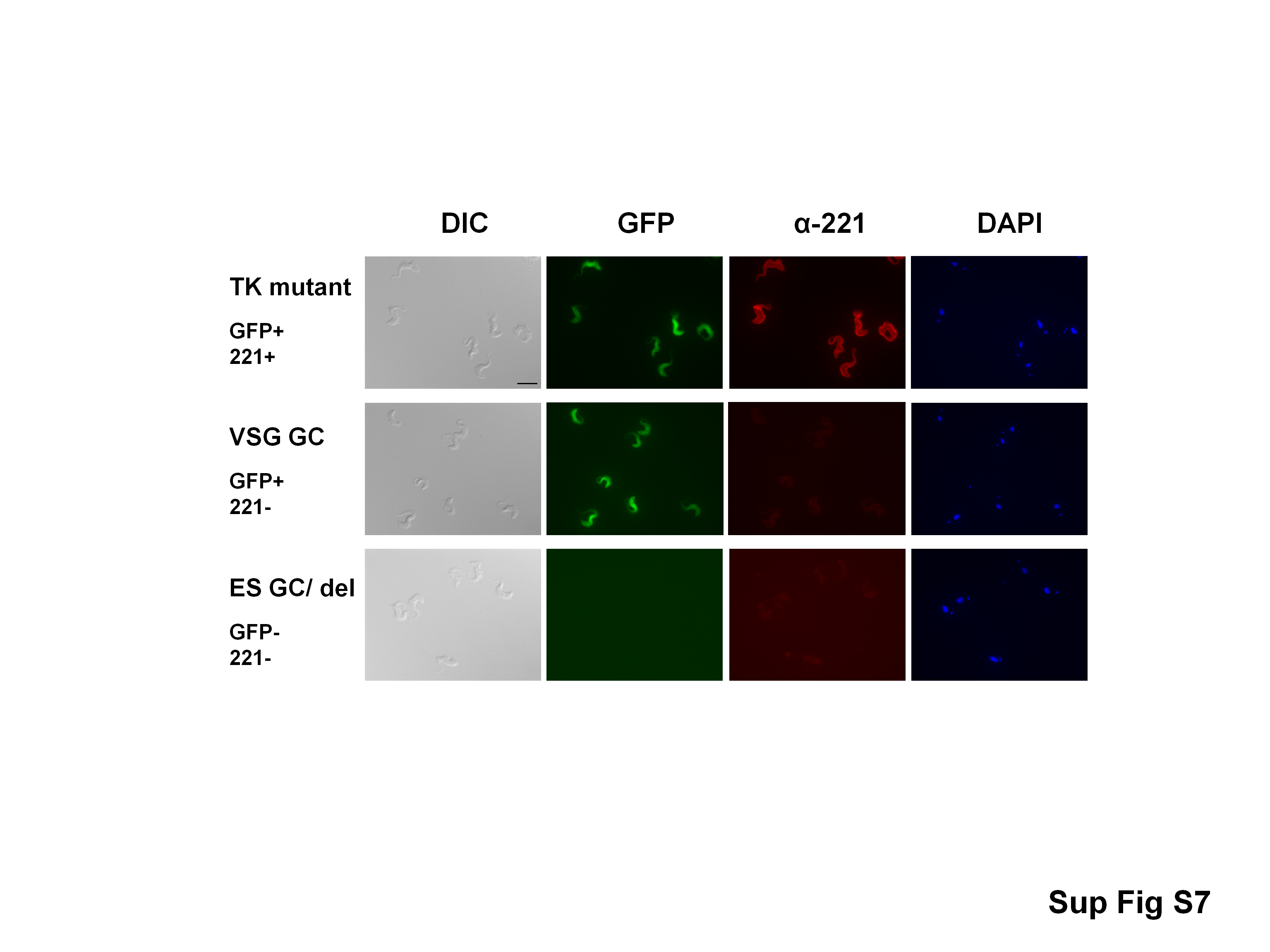

Supplement: Figure S7 — Analysis of VSG switching using fluorescence microscopy. Fixed T. brucei was subjected to immunofluorescence using an anti-VSG221 antibody. Cells were also monitored for GFP fluorescence, and DNA stained with DAPI. A differential interference contrast (DIC) image is shown for reference. For each channel, a similar exposure time was used to image the different clones. Scale bar is 10 µm. (TIF) [file ppat.1003010.s007.tif]
